# Supplementary material for: Deaggregation of mutant Plasmodium yoelii de-ubiquitinase UBP1 alters MDR1 localization to confer multidrug resistance
Source: Nat Commun. 2024 Feb 27;15:1774. doi: 10.1038/s41467-024-46006-3 (PMC10899652; doi:10.1038/s41467-024-46006-3)
Supplement: Supplementary file 1 — Supplementary Information [file 41467_2024_46006_MOESM1_ESM.pdf]

Supplementary information

Deaggregation of mutant *Plasmodium yoelii* de-ubiquitinase UBP1 alters MDR1 localization to confer multidrug resistance

Ruixue Xu[1,#], Lirong Lin[1,#], Zhiwei Jiao[1], Rui Liang[1], Yazhen Guo[1], Yixin Zhang[1], Xiaoxu Shang[1], Yuezhou Wang[1], Xu Wang[1], Luming Yao[1], Shengfa Liu[1], Xianming Deng[1], Jing Yuan[1,*], Xin-zhuan Su[2,*], Jian Li[1,*]

1. Supplementary Figures 1-9 and figure legends

2. Uncropped agarose gels for Supplementary Fig. 6d

3. Supplementary Tables 1-2

Supplementary Fig. 1

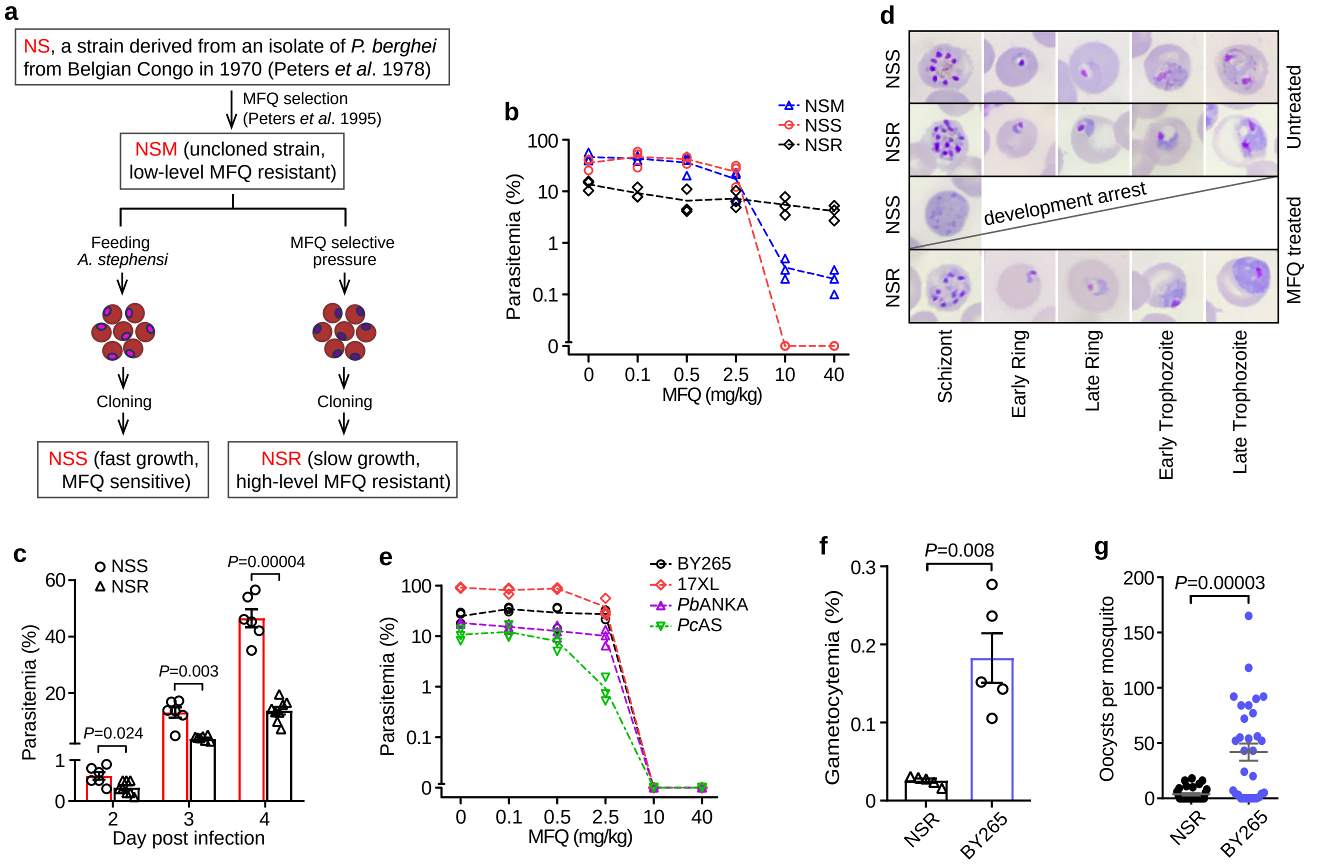

Supplementary Fig. 1. The origin, drug selection, and phenotypic characterization of *Plasmodium yoelii* NSS and NSR parasites.

(**a**) A diagram showing the origins and cloning of NSS and NSR parasites from *P. yoelii* NS and NSM.

(**b**) Parasitemia from ICR mice infected with *P. yoelii* NSM, NSS, and NSR and treated with different dosages of mefloquine (MFQ). $n = 3$ mice for each group.

(**c**) Mean parasitemia in Balb/c mice infected with NSS and NSR without MFQ treatment, showing significant growth reduction of the NSR parasite. Mean ± SEM from 6 mice in the NSS group, and 8 mice in the NSR group; two-tailed *t*-test.

(**d**) Morphology of parasite developmental stages of NSS and NSR parasites with or without 40 mg/kg MFQ treatment. Images were taken with a 100x oil microscope objective.

(**e**) Parasitemia from ICR mice infected with *P. yoelii* BY265, *P. yoelii* 17XL, *Plasmodium berghei* ANKA, and *Plasmodium chabaudi* AS after treatment with different dosages of MFQ. Three mice were used for each dosage in each group.

(**f**) Gametocytemia of *P. yoelii* BY265 and NSR parasites. Gametocytemia (%) was estimated as the proportion of all gametocytes among iRBCs. Mean ± SEM from 5 mice in each group; two-tailed *t*-test.

(**g**) The plot of numbers of oocysts from midguts of mosquitoes infected with *P. yoelii* BY265 and NSR parasites. Each dot represents oocyst counts from each mosquito midgut; mean ± SEM from 31 mosquito midguts in each group, two-tailed *t*-test.

Supplementary Fig. 2

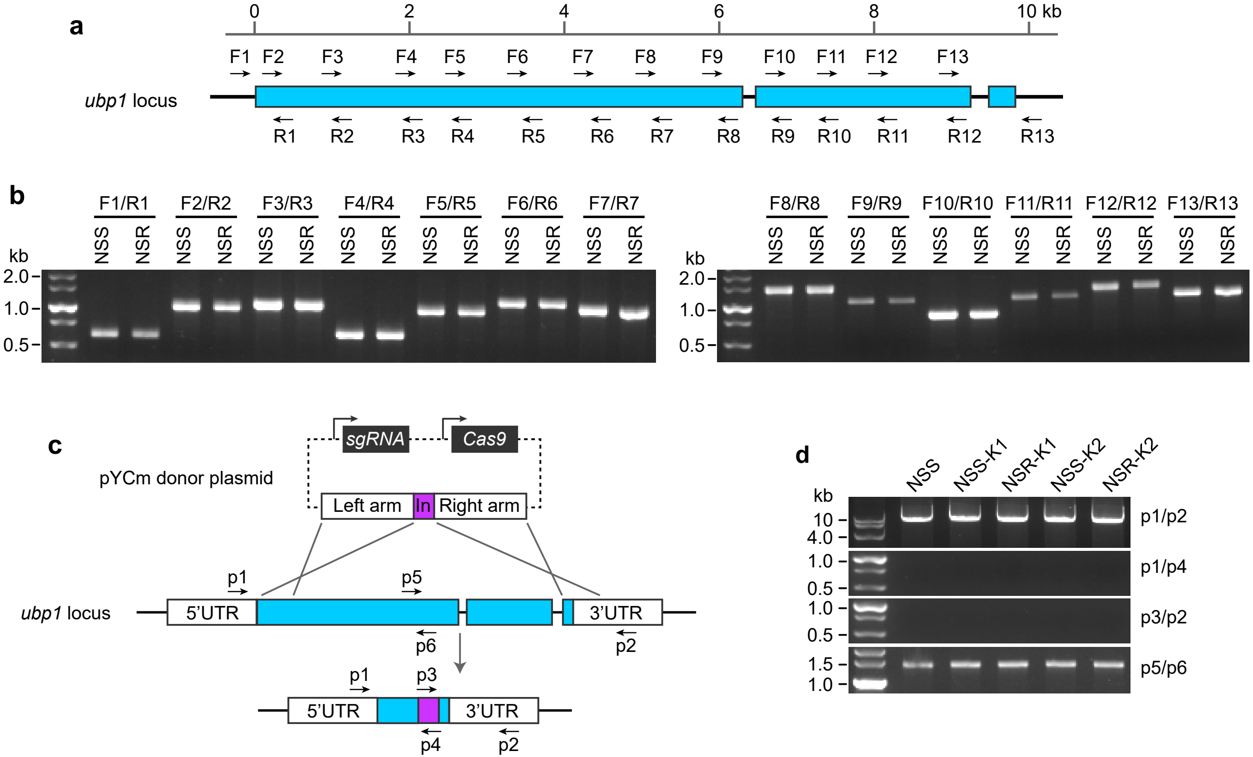

Supplementary Fig. 2. PCR amplification and sequencing of the gene encoding *P. yoelii* ubiquitin carboxyl-terminal hydrolase 1 (UBP1) and disruption of *ubp1* gene using the CRISPR/Cas9 technique.

(**a**) Diagram of the *ubp1* locus and primers for PCR amplification of the *ubp1* gene.

(**b**) Agarose gels showing PCR products amplified from genomic DNA of the *P. yoelii* isogenic lines NSS and NSR.

(**c**) Diagram showing a plasmid map with left arm from 5' coding region and right arm from a junction region between 3' coding and 3' untranslated (3'UTR) of *ubp1* gene as homologous DNA templates for integration into the *ubp1* locus.

(**d**) PCR products amplified from NSS and uncloned parasites of NSS or NSR transfected with the plasmid. The primer sequences used for PCR assays are listed in Supplementary Data 5.

Supplementary Fig. 3

```
Pf3D7     MSHINYNVEKRKSLKKHNNNNNNNNNIYNNKIDTPNIKNYDDSSKHINTNPQVLDSILLSNMEKDKKLKLLNNYINMFDKNKNDKVTTNHPSHNIYNRKNNDTYDDQDKDEQYVDTDDSF 120
PcAS      ------------------------------------------------------------------------------------------------------------------------ 0
PbANKA    ------------------------------------------------------------------------------------------------------------------------ 0
BY265     ------------------------------------------------------------------------------------------------------------------------ 0
17XL      ------------------------------------------------------------------------------------------------------------------------ 0
NSS       ------------------------------------------------------------------------------------------------------------------------ 0
NSR       ------------------------------------------------------------------------------------------------------------------------ 0

Pf3D7     SLSNTKKKINKRDIISYDNYIFEDEDKVSSKYLEYKNDSTSHMKKKKDEGSNRKGNINMDSNNNDDNNNNINMKSNTNNNNNNNNNNNKDDDYYDDNNYYHKNHSDSINNSINYNDIHRK 240
PcAS      ------------------------------------------------------------------------------------------------------------------------ 0
PbANKA    ------------------------------------------------------------------------------------------------------------------------ 0
BY265     ------------------------------------------------------------------------------------------------------------------------ 0
17XL      ------------------------------------------------------------------------------------------------------------------------ 0
NSS       ------------------------------------------------------------------------------------------------------------------------ 0
NSR       ------------------------------------------------------------------------------------------------------------------------ 0

Pf3D7     EKKNKKNTHNEKKYISNMYNFQYDDYDKKKKKKNTLETYDSDTKNSDIFINTGFLPYSLNKKKNNKKRKGKKKNEQEENRHNVNYDDNMDDDDDDNNDNNNDDDSNNNNNDDDSNNNNND 360
PcAS      -------------------------MDKNKKKNKKLEIRDPSDEIN-----------------NCEGEKGGTQF-----DESFPFD-----NPEPNHINEN------------------- 49
PbANKA    -------------------------MEKKKKKHKKREISESDNNMN-----------------NCGKEKGSIQH-----DELYNIEQYLSDNLESNNIIEN------------------- 54
BY265     -------------------------MEKKKKKPKERGITESDNNIN-----------------NCEKEKGSIQH-----GELYNIEQYLSDNLEPNNIIENAENAEN------------- 60
17XL      -------------------------MEKKKKKPKERGITESDNNIN-----------------NCEKEKGSIQH-----GELYNIEQYLSDNLEPNNIIENAENVEN------------- 60
NSS       -------------------------MEKKKKKHKERGITESDNNIN-----------------NCEKEKGSIQH-----GELYNIEQYLSDNLEPNNIIENAENAEN------------- 60
NSR       -------------------------MEKKKKKHKERGITESDNNIN-----------------NCEKEKGSIQH-----GELYNIEQYLSDNLEPNNIIENAENAEN------------- 60
                                    :*:*** :     : . : .                 *   .**  :       .    :     : : *:  :*

Pf3D7     DDSNNNNNDDDSNNNNNDDDSNNNNNDDSNNNNNDDSNNNNNDDSNNTGSFFKNKMIQSHVINNKYDNTNDYLDDLESFEYNKTKKKKKKKNDTIDDVFKNKRNDNTKIKYNYNNNNNNN 480
PcAS      -----------------------------------------------------------HNVN------NDDQNHVNIK------------KKNTSNSF--------------------E 72
PbANKA    -----------------------------------------------------------QNID------NDYQKHITIK---K-KNCNEKRGKNLSNSF--------------------E 85
BY265     --AENAENA---------------------ENAENVEN--------------------VENID------SDYQKDITIK---K-KKYNEKRGKKLINSF--------------------E 107
17XL      --AENAENAENV------------------ENVENVEN--------------------VENVD------SDYQKDITIK---K-KKYNEKRGKKLINSF--------------------E 110
NSS       --AENKNVDNKNAENKNVDN------KNAENKNVDNKN--------------------VENVD------SDYQKDITIK---K-KKYNEKRGKNLINSF--------------------E 122
NSR       --AENKNVDNKNAENKNVDN------KNAENKNVDNKN--------------------VENVD------SDYQKDITIK---K-KKYNEKRGKNLINSF--------------------E 122
                                                                     . ::      .*  ..:                ..  : *                    :

Pf3D7     NNVLEYELNYVHNSFDTHPKKWSHHSLPDNYEGEKKKKKNKTKKKRHDMNFYSIDKNNLDEQDLFSNQEALTILKNFAKENNVSHPYKEK-KINKNKKTSSNSMYKDYSNNINNYNNNND 599
PcAS      NDILEF----LPPNVDNNSDIFHAN-----------------------------EVNKIEETNLND-NEQNTMNIKNKDPNNRLDPKGND-------DNIVKNVESDFEK---------- 141
PbANKA    DNTLEF----LPQNVDDNSNNFQKT-----------------------------EINKIEENNLNS-TEAIKMNKKKKDINNRSDSKDNDCKCYDNDNADVKHTENDFEKNGNHSTNEND 171
BY265     DNTLEF----LPQNVDDNSNNFHKT-----------------------------EVNKIEKNNLNS-PEAIKMNKKNKDINHLSDSKDNDCKGQDNNNDDVKHTENNFEKNEN------- 186
17XL      DNTLEF----LPQNVDDNSNNFHKT-----------------------------EVNKIEKNNLNS-PEAIKMNKKNKDINHLSDSKDNDCKGQDNNNDDVKHTENNFEKNEN------- 189
NSS       DNTLEF----LSQNVDDNSNNFHKT-----------------------------EINKIEEDNLNS-AEAIKINKKNKNINHLSDSKDNGCKGQDNNNDDVKHTENNFEKNENNSTNEND 208
NSR       DNTLEF----LSQNVDDNSNNFHKT-----------------------------EINKIEEDNLNS-AEAIKINKKNKNINHLSDSKDNGCKGQDNNNDDVKHTENNFEKNENNSTNEND 208
          :: **:    :  ..* : . :                                : *:::: :*.   *  .:  :  . *:  .   :        .   :   .::.:

Pf3D7     ENNNYKDIVSN----YDNNYEYDKKNKNKIDVEQ---NVHM-------INYINVNNKQTNINNHNDNNFNVNKELSINDNVYENFIREYKNLQSLFSYNKNKIEDHFNPLTRIIEKNKED 705
PcAS      -NNNLLVTDENVNDHFENTNEQLNKNQDKDE--------------N-----IGKPNKREDS----DEHESGRKKRSVKDNVYEDFIREYKNLQSLFSYNQTMIKESFNPMPTILEDDNNY 237
PbANKA    KNKDLLAIEENINDHFESSDGYENEHSNKSEKGDEYENEHSNKSEKGDKPENEHSNKSTEM----NEHGNDGKKRSVKDNVYEDFIREYKNLQSLFAYNKTIIKENFNPMPTISEENKNG 287
BY265     -NKDILAIEENINHD---------------------------------------SNKTEEI----YESGNHGKKRSVKDNVYEDFIREYKNLQSLFAYNKNIIKENFISIPTISDEDKTG 262
17XL      -NKDILAIEENINHD---------------------------------------SNKSEEI----YESGNHGKKRSVKDNVYEDFIREYKNLQSLFAYNKNIIKENFISIPTISDEDKTG 265
NSS       KNKDILAIEENINHD---------------------------------------SNKSEEI----YESGNHGKKRSVKDNVYEDFIREYKNLQSLFAYNKNIIKENFNPIQTISDQDKTG 285
NSR       KNKDILAIEENINHD---------------------------------------SNKSEEI----YESGNHGKKRSVKDNVYEDFIREYKNLQSLFAYNKNIIKENFNPIQTISDQDKTG 285
           *::     .*                                            **  :      :  .  *: *::*****:************:**:. *:: *  :  * :.::

Pf3D7     NIVLENNINK-FILNAHEGLSKK-----------MLSYH--------------------------MDEQEDVQGMK------------SI-EDDNKGGDN----KGGDNKGGDKKGGDNK 770
PcAS      SDKNGSERNEDMKIPFDENVDKNATPRNHINPDYSAAYNLND--DAVISDVNKTSEKVSEEVHETMDNKVDETDHPHFSNQLNYPTNNDVQEDENKSLLRERGNENDKENGVETQNNDTN 355
PbANKA    SDIKMDKK---------------------------------------------------------MNKTEERNNHSDFSNQLNYPTNNNLKDDENKSRLSEQDDE------IEEKSSDNN 344
BY265     GDVKTVEQAG-----------------KQTDEDAGKQTDEDAGKQTDEDAGKQTDEDAGKQTEEDADETDEKNNDSDFSDQVNYPTNNNLKEDENKRCLSEKDDE------IEKKNSDNN 359
17XL      GDVKTVEQAG-----------------KQTDEDAGKQT----------------DEDAGKQTEEDADETDEKNNDSDFSDQVNYPTNNNLKEDENKRCLSEKDDE------IEKKNSDNN 346
NSS       GDVKTVQQSG-----------------KHID--------------------------------EDADETDKINNDSDFSDQVNYKTNDNLKEDENKRCLSEKDDE------IEKKSSDNN 350
NSR       GDVKTVQQSG-----------------KHID--------------------------------EDADETDKINNDSDFSDQVNYKTNDNLKEDENKRCLSEKDDE------IEKKSSDNN 350
          .     :                                                           ::  .  .              .: :*:**        :       : :..*.:

Pf3D7     GGDKKGDDK--KGDDNKDDDNDDDDDDDDNDDDDDDESTISYSKSDLSKIVEYINNDDMEEMTRFSNNNSVLQKKKQNKKKK-NEQNKD------NILTKN------------------- 862
PcAS      LGEEKEHMELKKEIKHKEFENQERK---QN--KEKEMNDASAPENE----------KNIDEISKK-KENHYIQKKNTIT--QLQEKKSSSNTSIASIINDNYDDHKISKKNNIEHVGKTQ 457
PbANKA    FGEGKEHEELKKEIKNKESENRENKK--IN--KQKETSDINAPESEENIILEK--EKDSDSISKK-KENLYMQKKNTNKLQKGKEQNSSSNISVASIINDNFDNYNISKNNSISKIDKTK 457
BY265     FGEDEELKE-------------------EN--KNKETSDVSVPENEENIILEK--EKDSDTISEK-KENIYIQKKNTNKLKKGHEQNSSSNISVASIINDNFDDYNI-----ISKIDKTK 450
17XL      FGEDEELKE-------------------EN--KNKETSDVSVPENEENIILEK--EKDSDTISEK-KENIYIQKKNTNKLKKGHEQNSSSNISVASIINDNFDDYNI-----ISKIDKTK 437
NSS       FGEGEELKE-------------------EN--KNKETSNVSVPENEENIILEK--EKDYDTISEK-KENIYIQKKNTNKLKKGHEQNSSSNISVASIINDNFDDYNI-----ISKIDKTQ 441
NSR       FGEGEELKE-------------------EN--KNKETSNVSVPENEENIILEK--EKDYDTISEK-KENIYIQKKNTNKLKKGHEQNSSSNISVASIINDNFDDYNI-----ISKIDKTQ 441
           *: :   :                    *  .:.: .  .  :.:          .: : ::.  ::*  :***:  .  : :*::..      .*:..*

Pf3D7     ---------------------SKHNSTHTIN----------CKNKK-------------------------------------------DLKNLSTSTNIMDESIHKNNNDNNMNSN--- 905
PcAS      FSNDSIKKKHHPIDRNEYKSSSPKNNRTNTEIKKGITESDNLKIKKIEKET----------------------------------------DDPNLYINILDENSK-------------- 523
PbANKA    TYNDLTKKKDGSIDNNEYKNDASKNYRPSIEINKSKNESDNIKIKKAEK------------------------------------QEGNKMNDHNFNINFLDESIK-------------- 527
BY265     TSSDLTKQ-----------NDASKNDRPSIEINKSKSEADNIKIKKAEKGAEKRPEKKAEKKAEKKAEKK----AEKKAEKKDEKKVENKMEDHNFNINILDESVK-------------- 541
17XL      TSSDLTKQ-----------NDASKNDRPSIEINKSKSEADNIKIKKAEKKAEKRPEKKAE----------------KKDEKKDEKKVENKMEDHNFNINILDESVKDNSKTDLFLNILDE 530
NSS       TSNDLTKQ-----------NDASKNDRPSIEINKSKSEADNIKIKKAEKKAEKKAEKKAEKKAEKRDEKKDEKKDEKKDEKKVENKMEDQNFNINILDESVK-------------- 536
NSR       TSNDLTKQ-----------NDASKNDRPSIEINKSKSEADNIKIKKAEKKAEKKAEKKAEKKAEKRDEKKDEKKDEKKDEKKVENKMEDQNFNINILDESVK-------------- 536
                               : :*   . :           * **                                             .: .   *::**. :

Pf3D7     --KNNNDNNM--N--SNNNNNDNNMNSNNNNNDNNMNSNN------N------NNNNYNSSSDMKIIESNNIPYSPNNNMKKKKKKKKKNLKEKLNNNIKYNEIITKTHIFPTNKIIQHD 1007
PcAS      ---DNKKKDLYLNNSFYLSKQDRSSKIQS------------------------AKNVEEVNGKIKKVKKHQASSDESSE-------------VNEMDHENYSKINKSLTEQHRKKDTKKK 603
PbANKA    ---DNSKTDLFLNISSDFSKHDNNDN----------------------DNKLKGVKNIEVNSQIKKTSKHRVIYDDNSEVDEM-----NQIDINLNDHENYNKINNNMNEQNRKGDIKKK 617
BY265     ---DNSKTDLFLNNSSDFSKHDNNDNSDSSDSS---DSSDSSDSGDSGDNKAKGGKNVEVNSKIKKPSEHRDIYDDNSQADET-----NQRDVNLNDNENYNKLNKNMNEQNRKGDIKKK 650
17XL      SVKDNSKTDLFLNNSSDFSKHDNNDNSDSSDSS---DSSDSSDSGDSGDNKAKGGKNVEVNSKIKKPSEHRDIYDDNSQADET-----NQRDINLNDNENYNKLNKNMNEQNRKGDIKKK 642
NSS       ---DNSKTDLFLNNSSDFSKHDNNDNSDNSDNSDSSDSGDSGDSGDSGDNKAKEGKNIKVNSKIKKISEHRDIYDDNSQADET-----NQRDINLNDNENYNKLNKNMNEQNRKGDIKKK 648
NSR       ---DNSKTDLFLNNSSDFSKHDNNDNSDNSDNSDSSDSGDSGDSGDSGDNKAKEGKNIKVNSKIKKISEHRDIYDDNSQADET-----NQRDINLNDNENYNKLNKNMNEQNRKGDIKKK 648
             :*...::  *     .::*.. :                             :  : ...:*  ..:.   . ..:              :  :: :*.:: ..      :   ::.

Pf3D7     KGVEY-----------ETTNSKHLLHKNINNIYNQSEQN---W-SLHEDLLKEVLTKEEYNEKLIKKNKNKNSKINKKTVDNKETHLHKQIAEKYDNIHTYIVETRKDKYSPSDHEKQNS 1112
PcAS      -KVEKKKKNNNNNT-ENTFDSKHLNDDNFSLSYFTE-DKKEGYDSIHEA--K--EVLQEYVNGKVK----------KHIRENKE-------TNKYKNVDPTLVKKIKNITKHTDFNINN- 698
PbANKA    -KVEKKKKNSNKNTFISN----HLNDDNISLSYFTEENKKEDYDSIHEI--E--KSSNEYIHVNNK----------KDIRDNKE-------TNKYKNIDPTIMKKIKNINKGTGLNINN- 710
BY265     KKVEKKKKNSNKNSFNLNDDN---DDDNISLSYFTEEDKKDDYDSNHEI--E--KSFNEYVNK-SK----------QNIRDNKE-------TNKYKNIDATIIKKIKNITKDADLNRNN- 744
17XL      KKVEKKKKNSNKNSFNLNDDNDDNDGDNISLSYFTEEDKKDDYDSNHEI--E--KSFNEYVNK-SK----------QNIRDNKE-------TNKYKNIDPTIIKKIKNITKDTDLNRNN- 739
NSS       KKVEKKKKNSNKNSFNLNDDDD--DDDNISLSYFTEEDKKDDYDSNHEI--E--KSFNEYVNK-SK----------QNIRDNKE-------TNKYKNIDPTIIKKIKNISKDTNLNRNN- 743
NSR       KKVEKKKKNSNKNSFNLNDDDD--DDDNISLSYFTEEDKKDDYDSNHEI--E--KSFNEYVNK-SK----------QNIRDNKE-------TNKYKNIDPTIIKKIKNISKDTNLNRNN- 743
            **             .        .*:.  *  . ::   : * **   :     :** .   *          :.  :***       ::**.*:.  :::. *:  . :. : :*

Pf3D7     FIKERVLHSKKKIKGKKNSKRNIKMVSRNKENKKERTMKSQ-----------NDNHLNNHESDDNNSIENSYEESAMYDEN------NSSIHDDNSKKEKFSDNEKYHE----RAEEEIV 1211
PcAS      -LDDQLCKLDPPI----------KHSKHHKETTKKKSVRQSSSSV-KKKHTKHSSKKNKKEDDPNDSDIKEWES-KFIEEDEEE---------------------------------DEE 772
PbANKA    ----RVCKLNSPIK-------EIKQVRNHKEIAKKKTLSPNSSFIKKKKHIKHSTKKNKKEDDANDSKIKKWES-KFVEENEEMEEENEEMEEENEEME--EENEEMEEENEEIEEEKEE 816
BY265     ----QTSKLNSTIK-------EIKQVKNHKEMTKKKALSQNSSFIKKKKNIKHSTKKNKKEDDTNDSEIKEWGS-KFVEEMEEKDEEE---------------------EEEEEEEEGEE 831
17XL      ----QVSKLNSTIK-------EIKQVRNHKEMTKKKALSQNSSFIKKKKNIKHSTKKNKKEDDTNDSEIKEWGS-KFVEEMEEKDEEE---------------------EEEEEEEEGEE 826
NSS       ----QTSKLNLTIK-------EIKQVRNHKEMTKKKALSQNSSFIKKKKNIKHSTKKNKKEDDTNGSEIKEWGS-KFVEEMEEEEEKEKE------------------KEEEEEEEEDEK 833
NSR       ----QTSKLNLTIK-------EIKQVRNHKEMTKKKALSQNSSFIKKKKNIKHSTKKNKKEDDTNGSEIKEWGS-KFVEEMEEEEEKEKE------------------KEEEEEEEEDEK 833
              :  : .  *          *   .:**  *::::  .           :..: *::*.* *.*  :.: .  : :*

Pf3D7     SDDLYQEDDNSDHSNK------------------------------------KIKMNMKSMTSFDKDKRRYTIQNLEEIKKKSKKSINKNENDKYGYNSDYMNDSGDFAVEKKDKKKKTQ 1295
PcAS      YNDPYLTIINEKQKKKESKHSKKKKIYMTNESFKNSISTVDDTQSGQEDDENDQAMQLSFFNFFDKQKNNQHIVNLQKEDN-------EIEK-------------DELNYKKKKK-KKKD 871
PbANKA    DNELYWETINEKKKKK-KKYSKKKKSQIMNESFNNSISSVDDIQSEQEEDENDKAMQLSFFNFFDKKK----------------------------------------------KKKKKH 889
BY265     DNELYWATINEKKKRKEKNCSKKRKSKIINESFNNSISSVEDIQSEQEEDENDKAIQLSFFNFFDKKKNNGNIVNIRKG---------EIER-------------EELNYKKKKKKKKEH 929
17XL      DNELYWATINEKKKRKEKNCSKKRKSKIMNESFNNSISSVEDIQSEQEEDENDKAIQLSFFNFFDKKKNNGNIVNIRKG---------EIER-------------EELNYKKKKKKKKEH 924
```

```
NSS     DNELYWATINEKKKRKEKKCSKKRKSKIMNESFNNSISSVEDIQSEQEEDENDKAIQLSFFNFFDKKKNNGNIVNIRKG---------EIER-------------EELSYKKKKKKKKEH  931
NSR     DNELYWATINEKKKRKEKKCSKKRKSKIMNESFNNSISSVEDIQSEQEEDENDKAIQLSFFNFFDKKKNNGNIVNIRKG---------EIER-------------EELSYKKKKKKKKEH  931
         :: *    *..:..*                                      .   :::.  :.  ***.*                                        * ** .

Pf3D7   QENCDNKYGNKYNKCDKDKDKDNYNNKDKFLP---SDQAFHYDNRKAKKKNKEDILKDQYNDEHIKEYFYSLIEGQVSKNNKN--------KKKKNSQRDYSLNKSTKEK----GVKKER  1400
PcAS    NR----------------KTNDILKDDVKFEHNINNFFSSFWDNENLKKKNKDDLYNLNNEN-NKDIYSVNKIEKKINKPYLHISKEDNINEQIDRLRREIAQNNILTEHDKHIDERKGD  974
PbANKA  SQ----------------KSNDILKENLKLENDINNFFISFWDNKNLKKNNKEDLYNFNNENNTKDIHSINKIEKKMSKQYIHTPKEDNLNEQIDILRKKNSQNNILQNQDKYIDEKKG-  992
BY265   SR----------------KSNDILKENFKFENNINNFFTSFWDNKK----NKENLYNFNNENNTKDIYSINKIEKKMSKQYAHILKEDNLNDQIDVIRTNYSQNNILRNREKCIDEKRG-  1028
17XL    SR----------------KSNDILKENLKFENNINNFFTSFWDNKK----NKENLYNFNNENNTKDIYSINKIEKKMSKQYVHISKEDNLNDQIDVIRTNYSQNNILRNRDKYIDEKRG-  1023
NSS     SR----------------KSNDVLKENLKFENNINNFFTSFWDNKK----NKENLYNFNNENNTKDIYPINKVEKKMSKQYVHISKEDNLNDQIDVIRTNYSQNNILRNRGKCIDEKKG-  1030
NSR     SR----------------KSNDVLKENLKFENNINNFFTSFWDNKK----NKENLYNFNNENNTKDIYPINKVEKKMSKQYVHISKEDNLNDQIDVIRTNYSQNNILRNRGKCIDEKKG-  1030
        ..                * :*  ::. *:     .    .:**.:    **::: : : ::   . :  . :* ::.*   :        .: .  : . : *:   ::   . ::

Pf3D7   LLHNKHFKETDSE--EDQNNKKNK------NNIYLK--KNYDQENEKDNEYENEKSYKKSTRPYYEEDHTPYRKQNIQDWSSYTKDKENKLNMDDDINMNKGNDQ--------------  1495
PcAS    IRKFLHWDDGKKGKHNGKDDKKEKDKLKEKDKIKYKEKMDYPNYEHNRNYDEDKRKHKKNHMENYDESYNPY---SIEEYNNYS-------------------------------------  1055
PbANKA  -------------KHSG----KDKDKFREKDKMKYRDKISYINYEHNRSYIDNKHKYKKNYLENYDELYN-Y---SIEEYNN---------------------------------------  1053
BY265   -------------KHNG----KDKDKFREKDRMKYRDKISYTN------YIDNKYKYKKNYLENHDQVYNNY---PIEEYDDDDDD-----------------------------------  1088
17XL    -------------RHNG----KDKDKFREKDRMKYRDKISYTN------YIDNKHKYKKNYLENHDQVYNNY---PIEEYDDDDD------------------------------------  1082
NSS     -------------KHNG----KNKDKFREKDRMKYRDKISYIN------YIDNKHKYKKNYLENYDQVYNNY---PIEEYDDDDDDDDDDDDDDDDDDDNNNNNNNNNNNNNNNNNNNNN  1124
NSR     -------------KHNG----KNKDKFREKDRMKYRDKISYIN------YIDNKHKYKKNYLENYDQVYNNY---PIEEYDDDDDDDDDDDDDDDDDDDNNNNNNNNNNNNNNNNNNNNN  1124
                      ..    *:*     :.:  :   .* :        ::: .:**.     ::: :. *    *:::..

Pf3D7   ---DVNRTYKNEKNKEE----DKYGKNEKNEKYDKYDKYEKYEKYDK------YKKDNKNQH---------DDPLYDNINKNYDNDNKGLEFFSNNFFHIKKFIEKKENENVHMSKIENS  1593
PcAS    --------------SSENELKRIYNKNEKK--YM--DGILLNKNYDEPINNSLKKKHHKHEDKENNYFLENNND-NSTAHNNNGYTTKGLGFFSSNFFHLKKLIEKKNVENNDSLKRENG  1156
PbANKA  ------NYSSSEHEKRENELKKIYNKNERK--YL--DGIILNKNYDESINNVLKKKDHKNEEKENNYFFQKNND-NSTVHNNSGYATKSLGFFSSNFFHLKRLLEKKNVETNDPLKRENE  1162
BY265   -----NNNYSSEDEKREKGFKKIYNKNEKK--YL--DGIILNKNYDEPINNVLKKKNHKNEEKENNNFFQKNNDNNSTVNNNSGYATKGLGFFSSNFFHLKRLLEKKNVETNDSLKRENE  1199
17XL    -----NNNYSSEDEKREKGFKKIYNKNEKK--YL--DGIILNKNYDEPINNVLKKKNHKNEEKENNNFFQKNNDNNSTVNNNSGYATKGLGFFSSNFFHLKRLLEKKNVETNDSLKRENE  1193
NSS     NNNNNNNNYSSEDEKREKRFKKIYNKNEKK--YL--DGIILNKNYDEPINNVLKKKDHKNDEKENNNFFQKNNDNNSTVNNNSGYATKGLGFFSSNFFHLKRLLEKKNVETNDSLKRENE  1240
NSR     NNNNNNNNYSSEDEKREKRFKKIYNKNEKK--YL--DGIILNKNYDEPINNVLKKKDHKNDEKENNNFFQKNNDNNSTVNNNSGYATKGLGFFSSNFFHLKRLLEKKNVETNDSLKRENE  1240
                      . *      *.***::  *   *     ::**:        **.:*::.          ::   ..  ::* .  .*.* ***.****:*:::***: *.  .  * **

Pf3D7   QKEE----------------ELNHKRNNLNSSGKTEKLEKFLGLYKENN-EAMDFYKSVLIEE--------NNSMNIS------------------------------------------  1646
PcAS    VDKFYEKNENNAKNEKKKLSSFDILQKFINNKG-DTKYGKIDDSYKRGNNDVAELYHISRRKGSINEYGDPHETAQISDGYSEKAGHNDIDEYTNFQTYRRNPDERDRRYYKDKREYHEN  1275
PbANKA  LDKFYEKN------EKKKSSSFDIFQKFINNKGHDMKCGKISNFYKRESHDYSDIYYTSRGKESFKECIDPNDTEQISDAYSEYIEHFDIDKYGNFRKYKRNKDDKNRKYYKDRHEYHEN  1276
BY265   LDKFYEKN------EKKKSSSFDIFQKLINNKTHDMKCEKMSNSYKRESNDYSDMYYTSREKENLKKYIYPNDTPQISDAYNDYIEHFGIDKYEKFQTYKGNKSDKDRTYYKDRCESHEN  1313
17XL    LDKFYEKN------EKKKSSSFDIFQKLINNKTHDMKCEKMSNSYKRESNDYSDMYYTSREKENLKKYIYPNDTPQISDAYNDYIEHFGIDKYEKFQTYKGNKSDKDRTYYKDRCESHEN  1307
NSS     LDKFYEKN------EKKKSSSFDIFQKLINNKTHDMKCEKMSNSYKRESNDYSDMYYTSREKENIKKNIYPNDTPQISDAYNDYIEHFGIDKYEKFQTYKGNKSDKDRKYYKDRYESHEN  1354
NSR     LDKFYEKN------EKKKSSSFDIFQKLINNKTHDMKCEKMSNSYKRESNDYSDMYYTSREKENIKKNIYPNDTPQISDAYNDYIEHFGIDKYEKFQTYKGNKSDKDRKYYKDRYESHEN  1354
         .:                  .::  :: :*..    *  *: .  **. .  :  ::*     :         ::: :**

Pf3D7   -------------------------------------------KNKINKNNIIDDRMKDNISKINRYNSDDTYIKVENNYDNKK-------EMNN---SDELNG-----NNNNNNNK---  1705
PcAS    EKH---------------------------EKNSKHKKHAEYKWKKINK-ELINDH----TDHI---EDDEKSVKFRKKFNYNLTRTGSNESMINEFGINKLNPQFSDEEKNENNNI---  1357
PbANKA  EKH---------------------------EKNSKHKKPAEYKCKKINK-ELLNDR----IHNI---EYDDKSLNFQKKYISNLTRTDSNETMVNEFSMDKLNHQSNNEDKNENNNDFNK  1361
BY265   GKNGKNGKNEKNEKNEKNEKNEKNEKSEKNSKNSKNKKRAECKSKKINK-ELLNDR----IQNI---EYDDKSLKFQKKYISNLTRTYSNEMLLNNFSVDKLDHQFNNGDENY-----EN  1420
17XL    GKNGKNEKNEKN---------EKNEKSEKNSKNSKNKKRAECKSKKINK-ELLNDR----IQNI---EYDDKSLKFQKKYISNLTRTYSNEMLLNNFSVDKLDHQFNNGDENY-----EN  1405
NSS     VKNGKNEKSEKI---------------EKSEKNSKNKKRAECKSKKINK-ELLNDR----IQNI---EYDDKSLKFQKKYISNLTRTYSNEMLVNNFSVDKLYHPFNNGDENC-----EN  1446
NSR     VKNGKNEKSEKI---------------EKSEKNSKNKKRAECKSKKINK-ELLNDR----IQNI---EYDDKSLKFQKKYISNLTRTYSNEMLVNNFSVDKLYHPFNNGDENC-----EN  1446
                                                  :**** ::::*:       :*    : *:.  ::..:::  :          : *    ::*        ::*

Pf3D7   -NNN----------NN----NNNNNNNN------NNNNNNNNINNGG------DKNRRNNFNN--N---NIYMCKNVKNIILSLELSNEEKINEVRKILFYSSSDEKKYIMNEILNILYI  1793
PcAS    ----------------------------------NKY----------------DEKDDEIYENSENFLYSVDNFKIVKNILLSLELTNDNKINKIRKILFYSSSEEKKKIMDEILNTLYI  1427
PbANKA  YENNNGFNKYENNNEFNKYENN---------NGFNKYENNNEFNKYENNNDLKKY--DEFYENSESFLYSVDNFKIVKNILLSLELTNDDKISKIRKILFYSPSEEKKKIMDEILNTLYM  1470
BY265   ------------------------------NENCENNENNENCENCENYENYENYE---NYENSESFLYSVDNFKIVKNILLSLELTNDNKISKIRKILFYSPSEEKKKIMDEILNTLYM  1507
17XL    CENYENYENY------------------------------------------ENYENNENNENSESFLYSVDNFKIVKNILLSLELTNDNKISKIRKILFYSPSEEKKKIMDEILNTLYM  1483
NSS     YENNENYENYENYENNENYENNENDENNENNENCENYENNENCENCENYENNENYENCKNNENSESFLYSVDNFKIVKNILLSLELTNDNKISKIRKILFYSPSEEKKKIMDEILNTLYM  1566
NSR     YENNENYENYENYENNENYENNENDENNENNENCENYENNENCENCENYENNENYENCKNNENSESFLYSVDNFKIVKNILLSLELTNDNKISKIRKILFYSPSEEKKKIMDENLNTLYM  1566
                                                     .       :*  .   .:   * ****:*****:*::**.::******* *:*** **:* ** **:

Pf3D7   YPQLYVSCIISLFYLFILDNEIFEKYFNADDLVYLFNEKIDFRYAEWFLKTYLFYKYKYSDNTHTKGSI-YYI-KKGSPRNSIKREDSNMYADQSVMAENIKKNYLNEGNQKDDDNKNNY  1911
PcAS    YPQLYVSCIICLFYLFILDNDLFERKFSPEDLVYLFNDKIDFRYAEWFLKTYLFYKYKFSNSSNSDDEHKSYTCDDELFDEPLKFKD------ASIGKDKAEK--LKNEQL---KNEKLK  1536
PbANKA  YPQLYVSCIICLFYLFILDNDLFERNFSSENLIYLFNPKIDFRYAEWFLKTYLFYKFKFSNSSNSDDGYNSYICENDSIGK-----------------EKVAK--IEKGR----------  1561
BY265   YPQLYVSCIISLFYLFILDNDLFERNFSSKDLVYLFNPKIDFRYAEWFLKTYLFYKFKFSNYSNSDDEHNSYICENSSVGK-----------------ENVAN--TEKGRIE-----RGR  1603
17XL    YPQLYVSCIISLFYLFILDNDLFERNFSSKDLVYLFNPKIDFRYAEWFLKTYLFYKFKFSNYSNSDDEHNSYICENSSVGK-----------------ENVAN--TEKGRIEKDRIEKGR  1584
NSS     YPQLYVSCIISLFYLFILDNDLFERNFYSKDLVYLFNPKIDFRYAEWFLKTYLFYKFKFSNYSNSDDEHNNYICGNNSVGK-----------------ENVAN--TEKGRIEKDR-----  1662
NSR     YPQLYVSCIISLFYLFILDNDLFERNFYSKDLVYLFNPKIDFRYAEWFLKTYLFYKFKFSNYSNSDDEHNNYICGNNSVGK-----------------ENVAN--TEKGRIEKDR-----  1662
        **********.*********::**: *  .:*:**** *****************:*:*: :::..     *   .    :                  ::  :   :: .

Pf3D7   DDKENNYDDKENNYDDKENNYDDNKNNYDDNKNNYDDKKNNYDDKKNNYDDNKNNYDYNNNKNDDDDSINVSSSLKGIHKNTFDPFFEKHSNNSLMDSGDDYLCDM-------------  2017
PcAS    SEKNGDINSLISEKDDTRGN-----------S-TLDG----------------------------IEGNEKTKRKNKAKNIICDNFFESYNS--SGDSMDNYVYCPNEKIEERNKN----  1610
PbANKA  IEKGGDINSLVSEKDDIEGN-----------K----------------------------------------KIQKIKNTIYDNFFESYNS--SGDSMDNYLYYQNDKIEEQNKI----  1623
BY265   IEKGEEANSLVSEKDDIEEN-----------E----------------------------------------KKQKIKNTIYDKFFESYSS--SGDSMDNYIYYKN---------GKNC  1660
17XL    IERGEEANSLVSEKDDIEEN-----------E----------------------------------------KKQKIKNTIYDKFFESYSS--SGDSMDNYIYYKNGK------NGKNC  1644
NSS     IERGEEANSLVSEKDDIEEN-----------E----------------------------------------KKQKIKNTIYDKFFESYSS--SGDSMDNYIYYKNCKIEEKNKNYKNG  1728
NSR     IERGEEANSLVSEKDDIEEN-----------E----------------------------------------KKQKIKNTIYDKFFESYSS--SGDSMDNYIYYKNCKIEEKNKNYKNG  1728
         ::  : :.  .: ** .  *           .                                         :  ::   * ***.:..     ** *:*:

Pf3D7   -----NNLSNNKKDIYILWTYFESSKCVGYNECKTLLSLCLKNENETCINNISASKLRSLVISIWSNIPSSKPKRSFIKLIFNWINNKKDDLHKKKNLFYLLKSEKKNNKNLSKICFNYF  2132
PcAS    ----NESDKQSERKIDEIWNKIESNKYVGYNECKMLLKICLTNENIQNIKMLSESKIRSLVISIWLNIRCSRPKKYFLKLIYNWINNKNDDFNKKKNLFNLLKAEKKHNKNITKACFNYF  1726
PbANKA  ----NESNKKLERKIDKTWNKIETNEYVGYNECKMLLKICLSNEQVQNIKLLSESKIRSLVISIWLNIRCSRPQKYFLKLIYNWINNKNDDFNKKKNLFNLLKAEKKYNKNITKACFNYF  1739
BY265   NDKNYEPNKELEKKIDKTWNKIEINEYVGYNECKMLLKICLSNEQIQNIKLLSESKIRSLVISIWLNIRCSRPKKYFFKLIYNWINNKNDNFNKKKNLFNLLKEEKKYNKNITKVCFSYF  1780
17XL    NDKNYEPNKELEKKIDKTWNKIEINEYVGYNECKMLLKICLSNEKVQNIKLLSESKIRSLVISIWLNIRCSRPKKYFFKLIYNWINNKNDNFNKKKNLFNLLKEEKKYNKNITKVCFSYF  1764
NSS     NDKNYEPNKELEKKIDKTWNKIETNEYVGYNECKMLLKICLSNEQVQNIKLLSESKIRSLVISIWLNIRCSRPKKYFLKLIYNWINNKNDNFNKKRNLFNLLKEEKKYNKNITKVCFSYF  1848
NSR     NDKNYEPNKELEKKIDKTWNKIETNEYVGYNECKMLLKICLSNEQVQNIKLLSESKIRSLVISIWLNIRCSRPKKYFLKLIYNWINNKNDNFNKKRNLFNLLKEEKKYNKNITKVCFSYF  1848
             :  .: ::.*   *.  :* .: ******* **.:**.**:   *: :* **:******** ** .*:*:: *:***:******:*:::**:*** *** *** ***::* **.**

Pf3D7   LNYLIKYKDNCSNDIIYILYLIDENELKIYSKNFIQNHKINFNQFISIWNIMCILFWDTDEINNFTFLQKN-KYYYYDFMLIFLKTFYDYINVNRDMREIMKMKLKRTFLTGYHHDVEEP  2251
PcAS    LKFLIKYKENCSNDIIYTLYLIDEHELKLYSKKFIEKNQININQFISIWNIMCILFWDTKKIHNFTLSNKNTNNYYYDFMLIFLKTFYEYIFTANSQFG-QNIYLKRMSNTSGVSAISFP  1845
PbANKA  LKFLIKYKENCSNDIIYTLYLIDENELKLYSKKFIEKNQININQFISIWNIMCILFWDTKKIHNFTFFKKNNNNYYYDFMLIFLKNFYEYIFPANSQIV-QNIYLKKTININSISTINLT  1858
BY265   LQFLIKYKENCSNDIIYTLYLIDENELKLHSKKFIEKNKININKFISIWNIMCILFWDTKKIHNFTFFKKNNNNYYYDFMLIFLKNFYEYIFPANPQIF-QNAYLKKTINFNSISTTNLI  1899
17XL    LQFLIKYKENCSNDIIYTLYLIDENELKLHSKKFIEKNKININKFISIWNIMCILFWDTKKIHNFTFFKKNNNNYYYDFMLIFLKNFYEYIFPANAQIF-QNAYLKKTINFNSISTTNLI  1883
NSS     LQFLIKYKENCSNDIIYTLYLIDENELKLHSKKFIEKNKININKFISIWNIMCILFWDTKKIHNFTFFKKNNNNYYYDFMLIFLKNFYEYIFPSNSQIF-QNAYLKKTINFNSISTTNLI  1967
NSR     LQFLIKYKENCSNDIIYTLYLIDENELKLHSKKFIEKNKININKFISIWNIMCILFWDTKKIHNFTFFKKNNNNYYYDFMLIFLKNFYEYIFPSNSQIF-QNAYLKKTINFNSISTTNLI  1967
        *::*****:******** ******:***::**:**::**:*:***************.:*:***: :** : ***********.**:**    .       :  **:     .      .

Pf3D7   SQEHMSLYQ-------EKN-------NIHNQDNRLSFTYMKKMSLSNSSINNKQDKHEDQNEYLNLFDIENIINNFNFTDFVNNEISRDNYFDSFFGATNMPIPSMSNISLAGNHTTQYE  2357
PcAS    NRNYNK--------------------------------LSSKASSTYA-ESQNKGNYNDESDDFNLFDVENIIKEFNFNDIVNAEVSSKNLMHS-PRDHNHDS-------HGTHPSESIE  1924
PbANKA  NQTHNQFSSKLS--------------------------------SSYP-ESKNKENYNDESDYFNLFDVENIIKDFNFNDISNIEVNTKNFTS-QFRDNNQDF-------IGTNHSETME  1937
BY265   NQPHNKPSSKMSSKMSSKMS--------SKMSSKLSSKLSSKMSSSYP----EPQNYNDESDNFDLFDVENIIKYFDFNDVSNIDINTKNFTSSQSRDNNPDS-------LGTNHSETIE  2000
17XL    NQPHNKPSSKISSKLSSKMSSKMSSKLSSKLSSKLSSKMSSKLSSSYP----EPQNYNDESDNFDLFDVENIIKYFDFNDVSNIDINTKNFTSSQSRDNNPDS-------LGTNHSETIE  1992
NSS     NQPHNKPSSKMSSKLSSKLSSKLSSKMSSKMSSKLSSKLSSKMSSSYL----EPQNYNDESDNSNLFDVENIIKYFSFNDVSNIDINTKNFTSSQSRDNNPDS-------LGTNHSETIE  2076
NSR     NQPHNKPSSKMSSKLSSKLSSKLSSKMSSKMSSKLSSKLSSKMSSSYL----EPQNYNDESDNSNLFDVENIIKYFSFNDVSNIDINTKNFTSSQSRDNNPDS-------LGTNHSETIE  2076
        .: : .                                         :       :   :::*:.:  :***:****: *.*.*.  * ::. .*          *          . : :   *

Pf3D7   KNTRHNYNSPLTHPLWRNR-----QEKERDLQRIKDEEERLKRRGGVPLTEAYDIENLIFLGICIKIVICRISNLLNAKSCLQQFHYFLNHKRLGLKIFKYSHIILVYFIPFFKKYYFLW  2472
PcAS    SA-----ENREEIEQAKENTIGIKNMLKFIIQKSK------KEKNMICCSDANELKLLVFLGICIKIVICRISNLLNAKACLEQFYYFINHKILGLKIFRHSHIVLVYFIPFFKKYYFLW  2033
PbANKA  SETRE-VEQSVHAKENTENTNGIKNMLKFIIQKIK------KEKNIISCVDINEIKMLIFLGICIKIVICRISNLLNSKTCLEQFYYFINHKILGLRIFKHSHIILIYFIPFFKKYYFLW  2050
BY265   SETREDAEQPGQAKENTENANGIKNMLKFIIQKIK------KEKNIISCVDINEIIMLVFLGICIKIVICRISNLLNSKTCLEQFYYFINHKILGLRKFKYSHIVLIYFIPFFKKYYFLW  2114
17XL    SETREDAEQPGQAKENTENANGIKNMLKFIIQKIK------KEKNIISCVDINEIIMLVFLGICIKIVICRISNLLNSKTCLEQFYYFINHKILGLRKFKYSHIVLIYFIPFFKKYYFLW  2106
NSS     SEIREDAEQPEQAKENTENTNGIKNMLKFIIQKIK------KEKNIISCVDTNEIIMLVFLGICIKIVICRISNLLNSKTCLEQFYYFINHKILGLRKFKNSHIVLIYFIPFFKKYYFLW  2190
NSR     SEIREDAEQPEQAKENTENTNGIKNMLKFIIQKIK------KEKNIISCVDTNEIIMLVFLGICIKIVICRISNLLNSKTCLEQFYYFINHKILGLRKFKNSHIVLIYFIPFFKKYYFLW  2190
        .       :.        :.      :  :  :*: *      *.:.  :    :  ::   *:*****************:*:**:**:**:*** ***: *: ***:*:*************

Pf3D7   KFIEHEIDKDIMNLIKYIMDHLENMQVENIPLSLCNI-----NNTSNQMLPGVSNQNMNERT--YAENLHNMNNIHNNKFCPSSYRHTQNILNMNSTHNNSSVNNNFNKMNHSISEKMGK  2585
PcAS    KFIEHEIDPEIIKLINAIINNLENLQNNKNINNIGSNPMLHPYTRSNTSLPEVDHFSFPSKDKNLNHTFNNFSTQYNPHDYKTSFSHSISIENNNSKQ------NFF-------------  2134
PbANKA  KFIEHEIDPEIIKLINAIINNLESLQINKNINNIENSPKFHPYIRSNTSLPKVDHLFFPSKDNKLNNIFSNFNMQSKPHNYKTSFSHSTSIESNN---------NNN-------------  2148
```

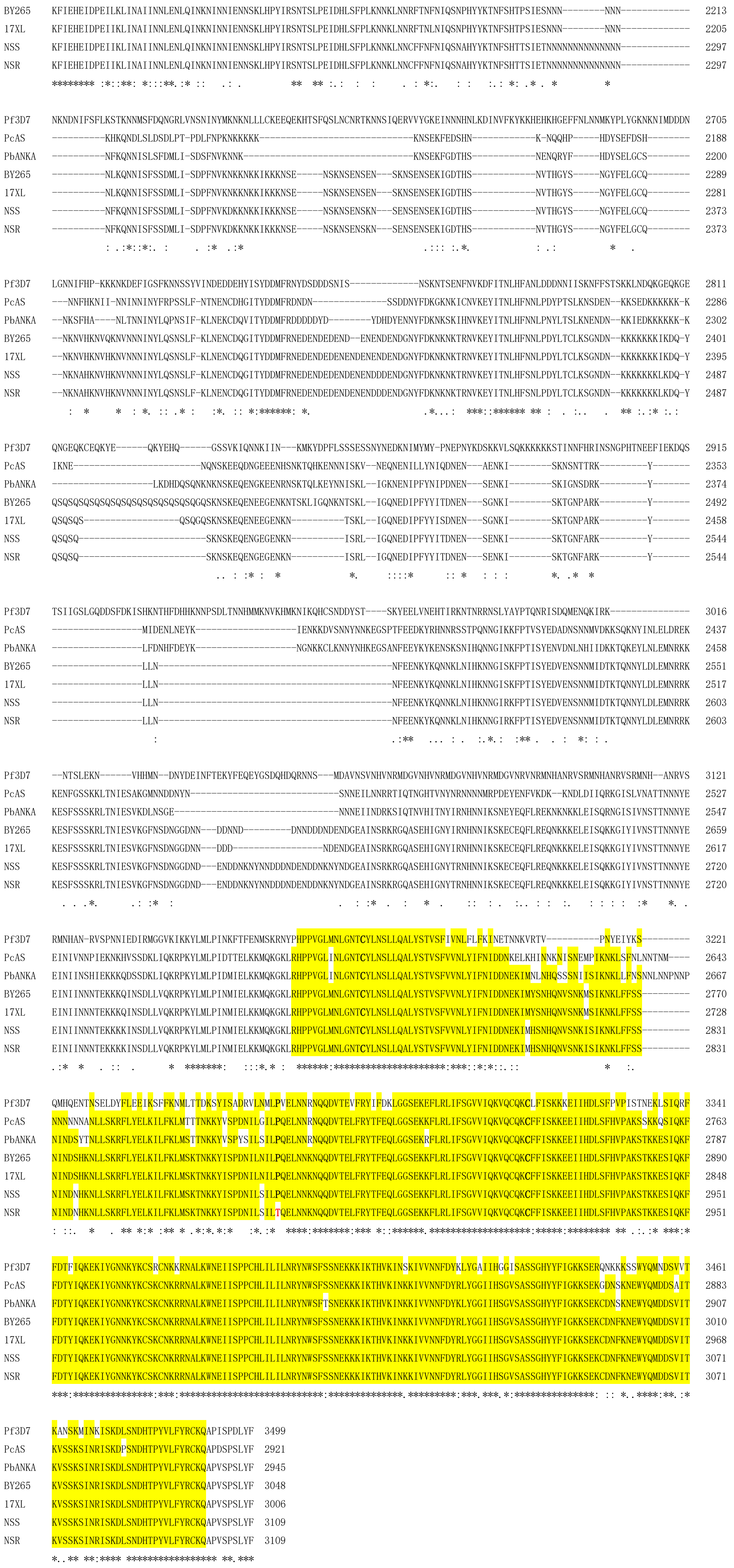

Supplementary Fig. 3. Alignment of the amino acid sequences of *Plasmodium* UBP1. Aligned sequences based on UBP1 proteins from *P. falciparum* 3D7 (PF3D7_0104300), *P. berghei* ANKA (PBANKA_0208800), *P. chabaudi* AS (PCHAS_0207200), *P. yoelii* 17XL (PY17X_0210200), BY265, NSS, and NSR. Primary sequence data for Pf3D7, PbANKA, PcAS, and Py17XL were downloaded from PlasmoDB (https://plasmodb.org/), sequences for *P. yoelii* BY265, NSS, and NSR were obtained based on the full coding region of the *ubp1* gene using the tiling sequencing strategy in this study. The CCR domain and UCH domain of UBP1 are highlighted in green and yellow, respectively. Conserved sites are indicated with the symbol '*'.

Supplementary Fig. 4

a

PBP1b *E. coli*	271	QLVKNLFLSSERSYWRKANEAYMALIMDARYSKDRILELYMNEVYLGQSGDNEIRGFPLA	330
PBP1a *E. coli*	124	QLARNFFLSPERTLMRKIKEVFLAIRIEQLLTKDEILELYLNKIYLGYRAYGVGAAAQVY	183
PBP1a *A. aeolicus*	121	QLAKNLFLTRERTLERKIKEALLAIKIERTFDKKKIMELYLNQIYLGSGAYGVEAAAQVY	180
NSS	1517	KIVKNILLSLELTNDNKISKIRKILFYSPSEEKKKIMDEILNTLYMYPQLYVSCIISLFY	1576
NSR	1517	KIVKNILLSLELTNDNKISKIRKILFYSPSEEKKKIMDENLNTLYMYPQLYVSCIISLFY	1576
17XL	1434	KIVKNILLSLELTNDNKISKIRKILFYSPSEEKKKIMDEILNTLYMYPQLYVSCIISLFY	1493
BY265	1458	KIVKNILLSLELTNDNKISKIRKILFYSPSEEKKKIMDEILNTLYMYPQLYVSCIISLFY	1517
PbANKA	1421	KIVKNILLSLELTNDDKISKIRKILFYSPSEEKKKIMDEILNTLYMYPQLYVSCIICLFY	1480
PcAS	1378	KIVKNILLSLELTNDNKINKIRKILFYSSSEEKKKIMDEILNTLYIYPQLYVSCIICLFY	1437
Pf3D7	1744	KNVKNIILSLELSNEEKINEVRKILFYSSSDEKKYIMNEILNILYIYPQLYVSCIISLFY	1803

Homology to glycosyltransferase domain of the bacterial Penicillin-Binding Proteins (PBPs)

b

```
Query   1    KKKIMDEILNTL   12
             K KIMDE+LN L
Sbjct   132  KQKIMDEVLNSL   143
```

ATP-binding cassette sub-family G member 1-like isoform X1 (*Belonocnema kinseyi*)

c

```
Query   2    KKIMDEILNT   11
             KKIMDEIL T
Sbjct   133  KKIMDEILKT   142
```

DNA repair protein (*Campylobacter concisus*)

Supplementary Fig. 4. Sequence alignment to bacterial Penicillin-Binding Proteins (PBPs) and a conserved UBP1 motif.

(**a**), Partial sequence homology of *Plasmodium* UBP1 amino acid sequence with bacterial Penicillin-Binding Proteins (PBPs) was predicted using the Swiss-Model automated server (https://swissmodel.expasy.org/) and aligned using Jalview (https://www.jalview.org/).

(**b**), Sequence alignment and partial homology to an ATP-binding cassette using BLAST searches.

(**c**), Sequence alignment to a motif in DNA repair protein using BLAST searches.

Supplementary Fig. 5

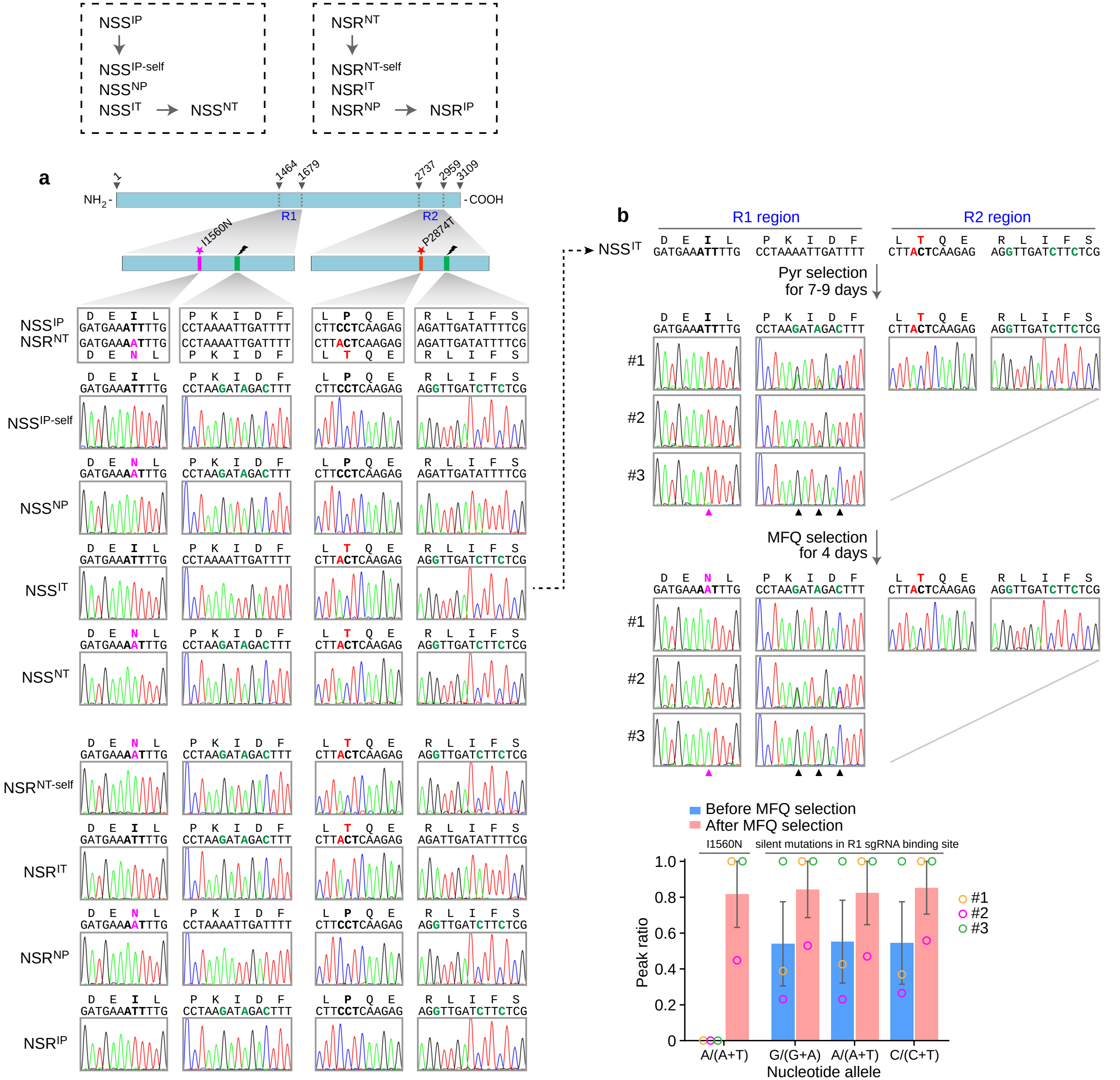

Supplementary Fig. 5. Allelic replacements of UBP1 mutations between the two *P. yoelii* isogenic lines NSS and NSR.

(**a**) Schematic diagram showing UBP1 protein with two regions being replaced and nucleotide sequences of allelic exchanged parasites. The numbers on the top of the protein bar are amino acid positions indicating start and end positions as well as the replacement regions. Partial nucleotide and amino acid sequences of nonsynonymous mutation (pink or red) between NSS and NSR, Cas9-gRNA targeting site (black arrowheads), and the silent mutation sites (green) to mutate the Cas9 cleavage site are presented within the regions. The electropherograms of DNA sequences showing changed nucleotides in the cross-replacement clones; the names of parasite clones are on the left.

(**b**) Electropherograms of DNA sequences for UBP1-targeted gene editing to introduce the I1560N mutation into the donor parasite NSS^{IT} that was used in the second round of transfection for generating the double replacement NSS^{NT} parasite. Transformed parasites were firstly subjected to Pyr selection (7 mg/L pyrimethamine, supplied in drinking water) for 7–9 days, and successful editing in the transfected parasites was observed after the additional selection with 20 mg/kg MFQ for 4 days. The electropherogram peaks of polymorphic nucleotides at three silent mutations between un-edited (A-T-T) and edited (G-A-C) parasites and at the I1560N mutation between NSS (T) and NSR (A) in the R1 region, showing sharply increased proportions from 0% to 44%–100% (NSS^{NT} over NSS^{NT}&NSS^{IT}) after the MFQ selection. #1, #2, and #3 were transformed parasites from three independent transfections.

Supplementary Fig. 6

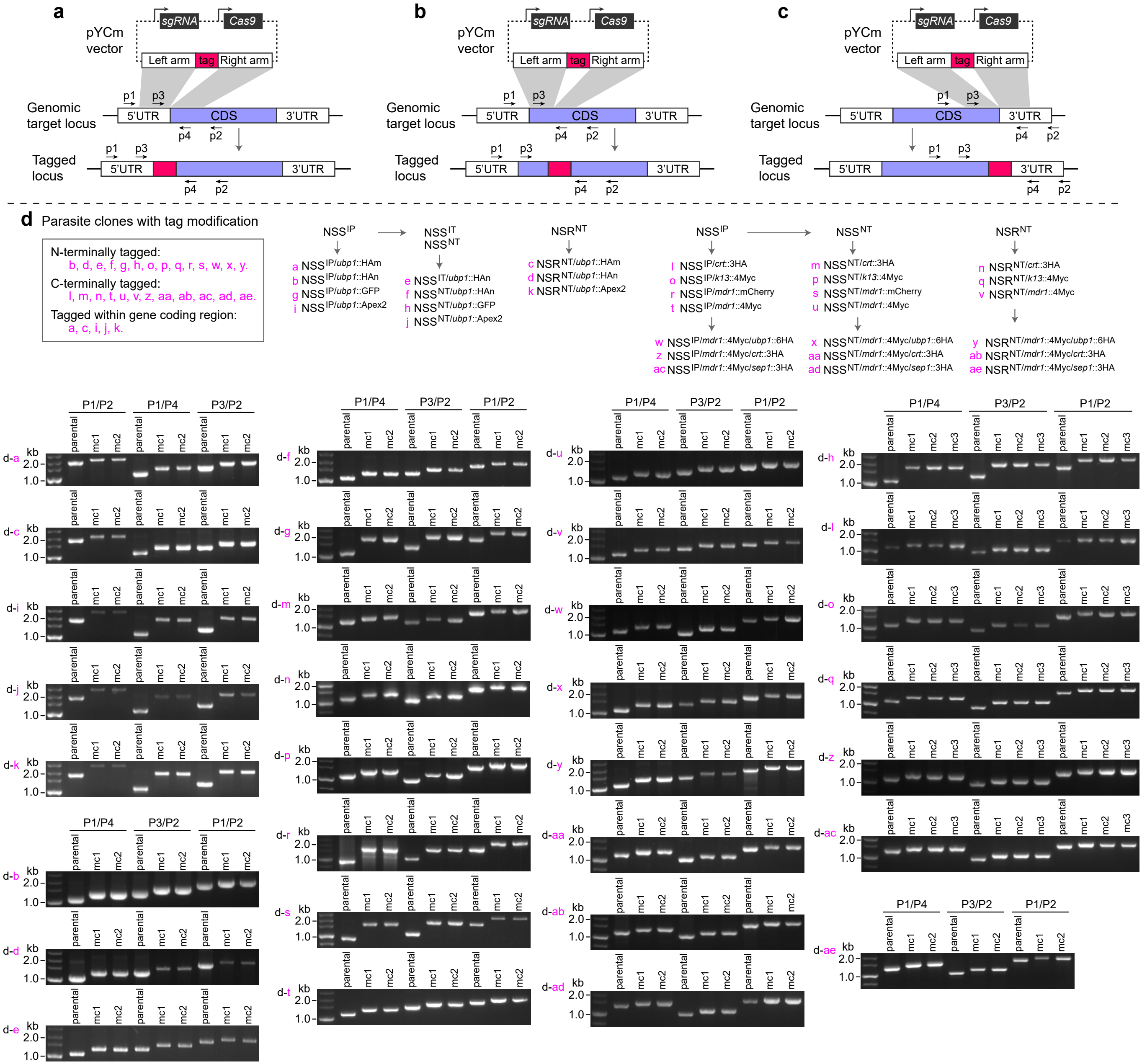

Supplementary Fig. 6. Summary of plasmid constructs and PCR confirmations of plasmid integration for gene tagging of modified parasites performed for this study.

(**a-c**) Schematics of CRISPR/Cas9-mediated editing strategy for tagging of an endogenous gene with 6HA, 4Myc, mCherry, GFP, APEX2, or 3HA sequence at the N-terminus (**a**), coding region (**b**), and C-terminus (**c**).

(**d**) Summary of generated recombinant parasite clones and agarose gels of correspondent confirmation PCR products. For each modification, the 5' and 3' integration were detected by diagnostic PCR using gene-specific primer pairs (Supplementary Data 5). Two to three modified clones (mc) were generally selected for further integration verification using DNA sequencing after the transfection, limiting-dilution cloning, and preliminary genotyping. (**d-a** to **d-aj**) are different recombinant parasites generated with matching agarose gels confirming correct integrations.

Supplementary Fig. 7

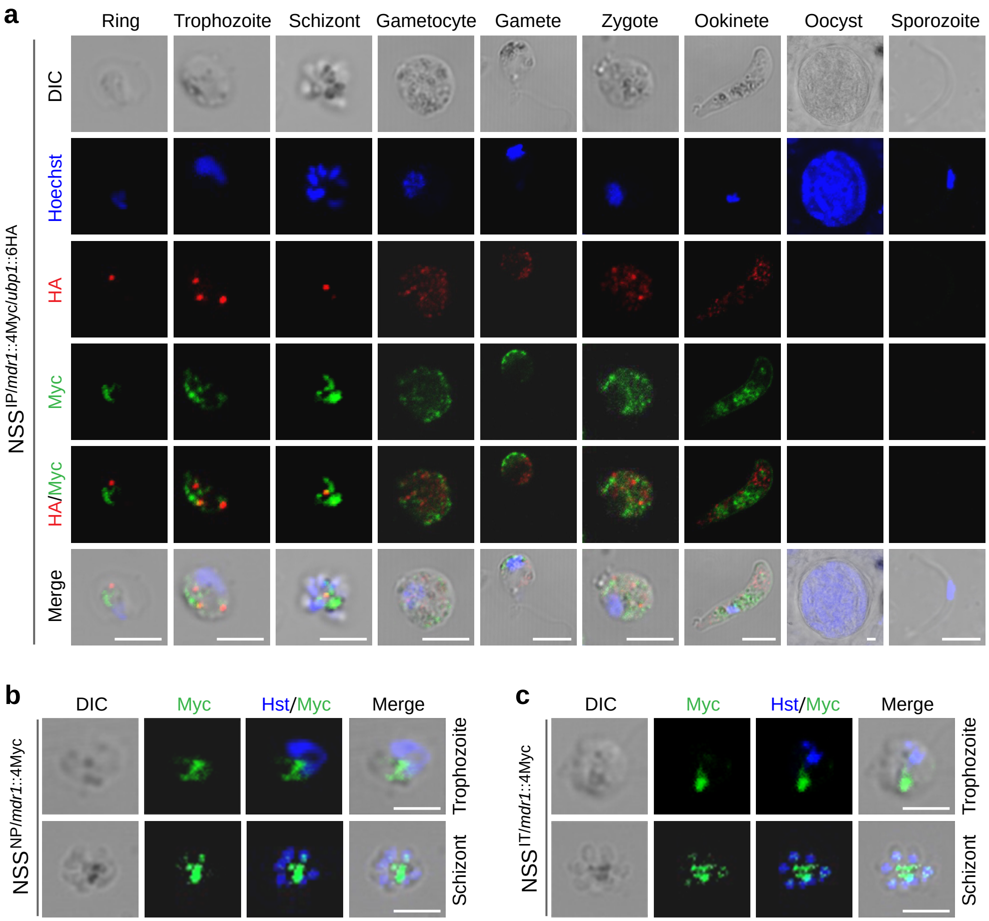

Supplementary Fig. 7. IFA analysis of *P. yoelii* UBP1 and MDR1 expressions during various developmental stages.

(**a**) Asexual blood stages (ring, trophozoite, and schizont), gametocyte, gamete, zygote, ookinete, oocyst, and sporozoite of the doubly tagged parasite $NSS^{IP/\mathit{mdr1}::4Myc/\mathit{ubp1}::6HA}$ were stained using anti-HA (red) and anti-Myc (green) antibodies.

(**b** and **c**) Trophozoite and schizont of the tagged parasites $NSS^{NP/\mathit{mdr1}::4Myc}$ (**b**), and $NSS^{IT/\mathit{mdr1}::4Myc}$ (**c**) using anti-Myc antibody. Hoechst 33342 (blue) is used for nuclear staining.

Supplementary Fig. 8

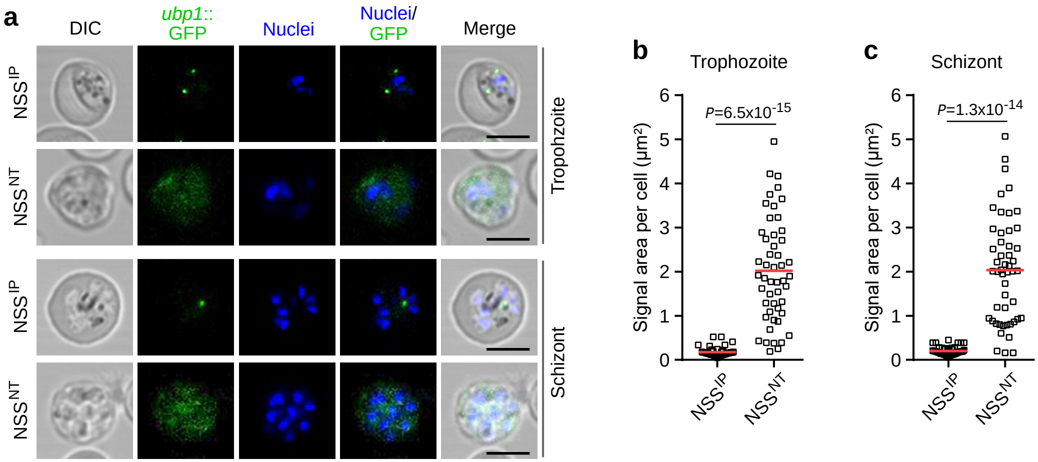

Supplementary Fig. 8. GFP tagging of UBP1 protein in NSS^{IP} and NSS^{NT} parasites.

(**a**) Representative images of trophozoite and schizont of modified parasites. Blue, Hoechst 33342 stain; Scale bars, 5 μm.

(**b** and **c**) Plots of quantitative analysis of UBP1::GFP signal area for trophozoites and schizonts, respectively. The horizontal lines show the mean values (n = 40 cells in each group), two-tailed t-test.

Supplementary Fig. 9

a

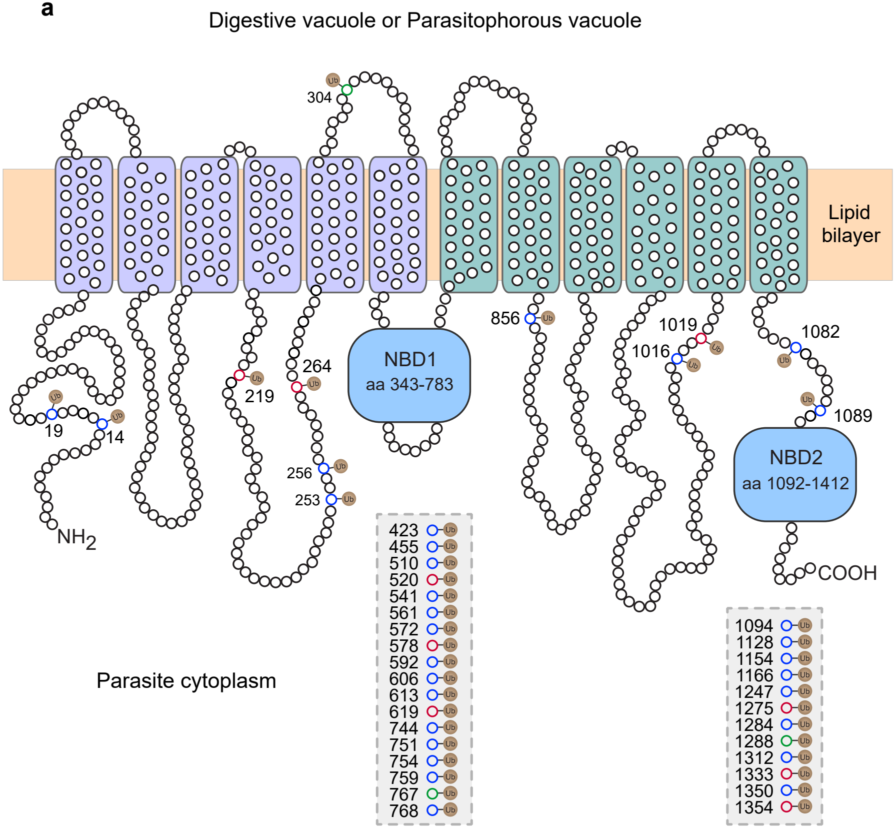

b

Lysine position	Peptide sequence
14	NNIKHEVEK
19	HEVEKELNK
219	KTSLLYNNNSMSIIEEALVGIK
253	TVASYCGESVILKK
256	FKLSEQFYSK
264	LSEQFYSKYMLK
304	IIINDIKNFNYANR
423	STILKLIER
455	SKIGVVSQDPLLFSNSIK
510	NGKCTSILDEISK
520	CTSILDEISKR
541	NTTSDLLEVISSINSVEDSKVVDVSK
561	VLIHDFVASLPDKYDTLVGSSASK
572	YDTLVGSSASKLSGGQK
578	LSGGQKQR
592	NPKILILDEATSYLDNK
606	ILILDEATSYLDNKSEYLVQK
613	SEYLVQKTINNLK
619	TINNLKGNENR
744	EKEIADTDKPTKPSFFK
751	EIADTDKPTKPSFFK
754	EIADTDKPTKPSFFK
759	EIADTDKPTKPSFFKR
767	EKKPPSNLSIVYK
768	KPPSNLSIVYK
856	NYYNNLIGEKVEK
1016	LIENAIDYDNKGEK
1019	LIENAIDYDNKGEKR
1082	LMSFKGDADK
1089	AKITFEK
1094	ITFEKYHPIMVR
1128	IEVKDVNFR
1154	KTTAIVGETGCGK
1166	TTAIVGETGCGKSTIMHLLMR
1247	ILLDGIDICDYNLKDLR
1275	FGKEDATLDDVK
1284	EDATLDDVKR
1288	VCKFAAIDEFIESLPNKYDTNVGPYGK
1312	YDTNVGPYGKSLSGGQK
1333	EPKILLLDEATSSLDSHSEK
1350	ILLLDEATSSLDSHSEKLIEK
1354	LIEKTIVDIK

Supplementary Fig. 9. Predicted structure of *P. yoelii* MDR1 and ubiquitinated lysine sites detected via quantitative LC-MS/MS analysis.

(**a**) Diagram showing the predicted *P. yoelii* MDR1 structure. The detected ubiquitination sites are marked with amino acid positions. Red circles, increased ubiquitination sites of *P. yoelii* NSR/NSS; green circles, decreased ubiquitination sites of *P. yoelii* NSR/NSS; blue circles, no change in signal.

(**b**) List of peptide sequences with ubiquitinated lysine (K) highlighted in red. NBD, nucleotide-binding domain.

Uncropped agarose gels for Supplementary Fig. 6c: Box shows the represented gel.

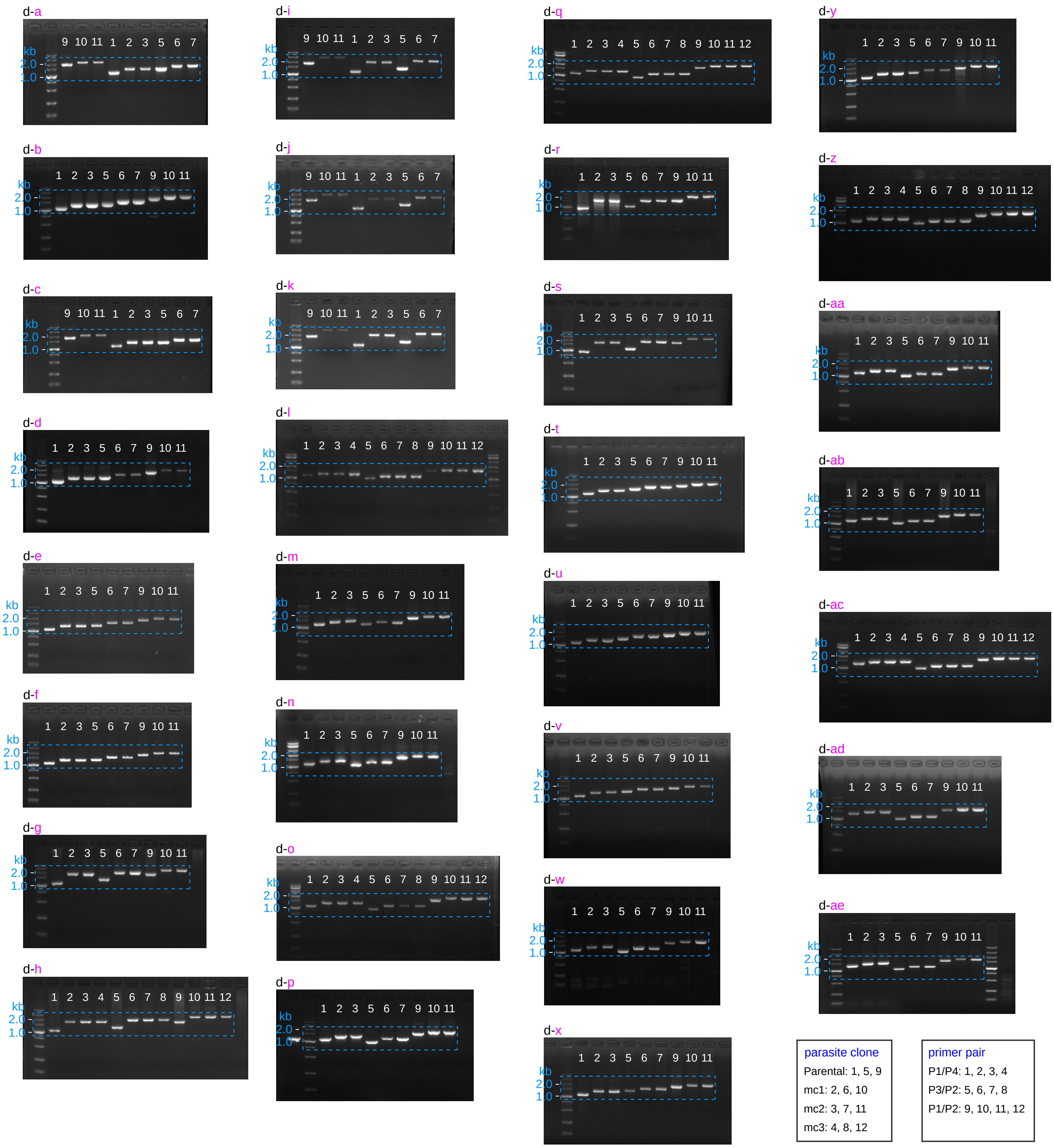

Supplementary Table 1. Nonsynonymous substitutions in genes detected by Illumina sequencing between *P. yoelii* isogenic lines NSS and NSR.

Chromosome	Position	17X Ref	NSS_seq	Alt_depth	NSR_seq	Alt_depth	Anno_Gene	Length (bp)	Anno_Pos	Gene direction	NSS Genotype	NSR Genotype	NSS aa	NSR aa	Confirmed by sanger sequencing	Product
Py17X_02_v3	445695	G	G	77	T	86	PY17X_0210200	9360	exonic	Reverse	CCT	ACT	Pro	Thr	yes	ubiquitin carboxyl-terminal hydrolase 1, putative
Py17X_02_v3	449778	A	A	77	T	60	PY17X_0210200		exonic	Reverse	ATT	AAT	Ile	Asn	yes	
Py17X_05_v3	173113	T	A	47	T	21	PY17X_0501400	8395	exonic	Forward	AAA	AAT	Lys	Asn	/	reticulocyte binding protein, putative (Py235)
Py17X_06_v3	962262	A	G	15	A	57	PY17X_0622500	2958	exonic	Reverse	TGT	GAC	Cys	Asp	an indel "GAAAAAAATTGC" was detected in NSR	zinc finger protein, putative
Py17X_12_v3	498501	A	A	76	G	117	PY17X_1211200	1602	exonic	Reverse	TTC	TCC	Phe	Ser	yes	tubulin-tyrosine ligase, putative
Py17X_12_v3	1993717	A	A	55	T	21	PY17X_1249900	8410	exonic	Reverse	ATT	AAT	Ile	Asn	/	reticulocyte binding protein, putative (Py235)
Py17X_12_v3	1994061	C	A	76	C	29	PY17X_1249900		exonic	Reverse	GAT	GAG	Asp	Glu	/	
Py17X_12_v3	1994090	G	A	73	G	22	PY17X_1249900		exonic	Reverse	TCA	CCA	Ser	Pro	/	
Py17X_12_v3	1995083	C	C	93	T	24	PY17X_1249900		exonic	Reverse	GAA	AAA	Glu	Lys	/	
Py17X_12_v3	1999352	A	A	78	T	19	PY17X_1249900		exonic	Reverse	TAT	AAT	Tyr	Asn	/	

Supplementary Table 2. *In vivo* IC_{50} and IC_{90} values of MFQ, LUM, PPQ, and DHA against parental *P. yoelii* NSS, NSR and allelic replaced parasites.

	Mean IC_{50} ± SEM (mg/kg)									
Drug	NSS^{IP}	$NSS^{IP\text{-}self}$	NSS^{NP}	NSS^{IT}	NSS^{NT}	NSR^{NT}	$NSR^{NT\text{-}self}$	NSR^{IT}	NSR^{NP}	NSR^{IP}
MFQ	2.55 ± 0.35	2.56 ± 0.03	3.09 ± 0.07	2.43 ± 0.35	/	/	/	3.00 ± 1.58	2.74 ± 0.25	2.87 ± 0.11
LUM	0.57 ± 0.15	0.78 ± 0.01	0.65 ± 0.15	0.76 ± 0.15	/	/	/	0.31 ± 0.02	0.88 ± 0.04	0.75 ± 0.16
PPQ	2.42 ± 0.43	2.32 ± 0.07	1.29 ± 0.23	2.50 ± 0.41	/	/	/	2.28 ± 0.04	2.43 ± 0.23	1.00 ± 0.11
DHA	0.57 ± 0.09	0.86 ± 0.24	0.60 ± 0.07	0.81 ± 0.44	0.25 ± 0.07	0.24 ± 0.09	0.13 ± 0.03	0.62 ± 0.44	0.57 ± 0.14	0.61 ± 0.15

	Mean IC_{90} ± SEM (mg/kg)									
Drug	NSS^{IP}	$NSS^{IP\text{-}self}$	NSS^{NP}	NSS^{IT}	NSS^{NT}	NSR^{NT}	$NSR^{NT\text{-}self}$	NSR^{IT}	NSR^{NP}	NSR^{IP}
MFQ	3.40 ± 0.42	3.27 ± 1.59	4.21 ± 0.08	4.78 ± 1.44	/	/	/	3.85 ± 1.21	3.66 ± 0.52	4.88 ± 0.84
LUM	0.68 ± 0.15	0.88 ± 0.01	1.23 ± 0.06	0.82 ± 0.12	/	/	/	0.65 ± 0.09	1.07 ± 0.04	0.92 ± 0.14
PPQ	2.98 ± 0.24	2.70 ± 0.09	3.24 ± 0.65	3.09 ± 0.11	/	/	/	2.77 ± 0.06	3.00 ± 0.13	2.51 ± 0.18
DHA	0.78 ± 0.39	1.53 ± 0.05	0.82 ± 0.16	1.34 ± 0.49	1.74 ± 0.44	1.12 ± 0.60	1.93 ± 0.36	0.86 ± 0.48	1.05 ± 0.28	2.13 ± 0.13

MFQ, mefloquine hydrochloride; LUM, lumefantrine; PPQ, piperaquine phosphate; DHA, dihydroartemisinin.
'/', No IC_{50} or IC_{90} can be calculated for these parasites due to limited redcution in parasitemia.
