# Supplementary material for: Deaggregation of mutant Plasmodium yoelii de-ubiquitinase UBP1 alters MDR1 localization to confer multidrug resistance
Source: Nat Commun. 2024 Feb 27;15:1774. doi: 10.1038/s41467-024-46006-3 (PMC10899652; doi:10.1038/s41467-024-46006-3)
Supplement: Supplementary file 3 — Description of Additional Supplementary Files [file 41467_2024_46006_MOESM3_ESM.docx]

**Description of Additional Supplementary Files**

**Deaggregation of mutant *Plasmodium* *yoelii* de-ubiquitinase UBP1 alters MDR1 localization to confer multidrug resistance**

Ruixue Xu^1,#^, Lirong Lin^1,#^, Zhiwei Jiao^1^, Rui Liang^1^, Yazhen Guo^1^, Yixin Zhang^1^, Xiaoxu Shang^1^, Yuezhou Wang^1^, Xu Wang^1^, Luming Yao^1^, Shengfa Liu^1^, Xianming Deng^1^, Jing Yuan^1,^*, Xin-zhuan Su^2,^*, Jian Li^1,^*

**1. Supplementary Data 1**. Microsatellite genotypes from uncloned progeny pools of the genetic crosses of *Plasmodium yoelii* BY265 x NSR.

**2. Supplementary Data 2**. Summary of proteins with ubiquitination sites in asexual blood stages of *P. yoelii* NSS and NSR.

**3. Supplementary Data 3**. List of proteins with differential ubiquitination sites in the asexual blood stages between *P. yoelii* NSS and NSR.

**4. Supplementary Data 4**. List of key reagents and materials used in this study.

**5. Supplementary Data 5**. Oligonucleotides and primers used in this study.
