# Supplementary material for: Deaggregation of mutant Plasmodium yoelii de-ubiquitinase UBP1 alters MDR1 localization to confer multidrug resistance
Source: Nat Commun. 2024 Feb 27;15:1774. doi: 10.1038/s41467-024-46006-3 (PMC10899652; doi:10.1038/s41467-024-46006-3)
Supplement: Supplementary file 9 — Reporting Summary [file 41467_2024_46006_MOESM9_ESM.pdf]

nature portfolio

Corresponding author(s): Jian Li, Xin-zhuan Su, Jing Yuan

Last updated by author(s): Feb 4, 2024

Reporting Summary

Nature Portfolio wishes to improve the reproducibility of the work that we publish. This form provides structure for consistency and transparency in reporting. For further information on Nature Portfolio policies, see our Editorial Policies and the Editorial Policy Checklist.

Statistics

For all statistical analyses, confirm that the following items are present in the figure legend, table legend, main text, or Methods section.

n/a	Confirmed	
☐	☒	The exact sample size (*n*) for each experimental group/condition, given as a discrete number and unit of measurement
☐	☒	A statement on whether measurements were taken from distinct samples or whether the same sample was measured repeatedly
☐	☒	The statistical test(s) used AND whether they are one- or two-sided<br>*Only common tests should be described solely by name; describe more complex techniques in the Methods section.*
☒	☐	A description of all covariates tested
☒	☐	A description of any assumptions or corrections, such as tests of normality and adjustment for multiple comparisons
☐	☒	A full description of the statistical parameters including central tendency (e.g. means) or other basic estimates (e.g. regression coefficient) AND variation (e.g. standard deviation) or associated estimates of uncertainty (e.g. confidence intervals)
☐	☒	For null hypothesis testing, the test statistic (e.g. *F*, *t*, *r*) with confidence intervals, effect sizes, degrees of freedom and *P* value noted<br>*Give P values as exact values whenever suitable.*
☒	☐	For Bayesian analysis, information on the choice of priors and Markov chain Monte Carlo settings
☒	☐	For hierarchical and complex designs, identification of the appropriate level for tests and full reporting of outcomes
☒	☐	Estimates of effect sizes (e.g. Cohen's *d*, Pearson's *r*), indicating how they were calculated

Our web collection on statistics for biologists contains articles on many of the points above.

Software and code

Policy information about availability of computer code

Data collection	Fluorescent images were captured using Zeiss LSM 780 confocal microscopy; Electron microscopical images were acquired using Hitachi HT-7800 electron microscope; Images for hemozoin quantification were captured by a reflection contrast polarized light microscopy (Olympus IX83); Libraries for genomic DNA seq were sequenced by Illumina HiSeq platform; Tryptic peptides were analyzed by Liquid chromatography-tandem mass spectrometry.
Data analysis	Fluorescent images were processed in ZEN 3.1 (blue edition); Fiji (ImageJ, win32) was used for image quantification analysis; mass spectrometry data were processed using MaxQuant search engine (v.1.5.2.8); Sequence reads from Illumina HiSeq were aligned to the P. yoelii 17X reference genome (https://plasmodb.org/) using Burrows-Wheeler Aligner, SNP sets were called using Samtools; Disordered regions were identified using PONDR (http://www.pondr.com/); Amino acid sequences were aligned with Clustal Omega (https://www.ebi.ac.uk/Tools/msa/clustalo/) or Jalview (https://www.jalview.org/); Statistical analysis was performed in Excel or GraphPad Prism (version 8.0.1).

For manuscripts utilizing custom algorithms or software that are central to the research but not yet described in published literature, software must be made available to editors and reviewers. We strongly encourage code deposition in a community repository (e.g. GitHub). See the Nature Portfolio guidelines for submitting code & software for further information.

Data

Policy information about availability of data

All manuscripts must include a data availability statement. This statement should provide the following information, where applicable:

- Accession codes, unique identifiers, or web links for publicly available datasets
- A description of any restrictions on data availability
- For clinical datasets or third party data, please ensure that the statement adheres to our policy

The genomic sequences of target genes and predicted protein sequences were downloaded from PlasmoDB database (http://plasmodb.org/). The sgRNA of target genes were identified using Tefor Infrastructure server (http://crispor.tefor.net/). The Illumina sequencing data is submitted to the Genome Sequence Archive in National Genomics Data Center (CRA013922). Mass spectrometry proteomics data are submitted to PRIDE (PXD047782, PXD047811).

Research involving human participants, their data, or biological material

Policy information about studies with human participants or human data. See also policy information about sex, gender (identity/presentation), and sexual orientation and race, ethnicity and racism.

Reporting on sex and gender	Not applicable
Reporting on race, ethnicity, or other socially relevant groupings	Not applicable
Population characteristics	Not applicable
Recruitment	Not applicable
Ethics oversight	Not applicable

Note that full information on the approval of the study protocol must also be provided in the manuscript.

Field-specific reporting

Please select the one below that is the best fit for your research. If you are not sure, read the appropriate sections before making your selection.

☒ Life sciences ☐ Behavioural & social sciences ☐ Ecological, evolutionary & environmental sciences

For a reference copy of the document with all sections, see nature.com/documents/nr-reporting-summary-flat.pdf

Life sciences study design

All studies must disclose on these points even when the disclosure is negative.

Sample size	Sample size was determined by reliability and robustness of phenotype based on our preliminary experiments and previous published work (Nair et al., Nature Communications 2017, 8(1):223; Li et al., Proc Natl Acad Sci USA 2011, 108(31):E374-82.). Proper positive and/or negative controls were applied for each experiment.
Data exclusions	No data was excluded from the analyses.
Replication	All key experiments were performed at least three different biological replicate and all replication attempts were successful.
Randomization	The mice and mosquitoes were always randomly allocated into control and experimental groups. For other biochemical experiments, samples were also randomly divided into experimental groups.
Blinding	For in vivo drug assays, following randomization, treatment groups were known. For all studies, blinding was not relevant because of objective readouts.

Reporting for specific materials, systems and methods

We require information from authors about some types of materials, experimental systems and methods used in many studies. Here, indicate whether each material, system or method listed is relevant to your study. If you are not sure if a list item applies to your research, read the appropriate section before selecting a response.

Materials & experimental systems

n/a	Involved in the study
☐	☒ Antibodies
☒	☐ Eukaryotic cell lines
☒	☐ Palaeontology and archaeology
☐	☒ Animals and other organisms
☒	☐ Clinical data
☒	☐ Dual use research of concern
☒	☐ Plants

Methods

n/a	Involved in the study
☒	☐ ChIP-seq
☒	☐ Flow cytometry
☒	☐ MRI-based neuroimaging

Antibodies

Antibodies used

mouse anti-Myc (cat#2276, Cell Signaling Technology (CST), 1:2,000 for Western blotting (WB), 1:8,000 for immunofluorescence (IF), 1:1,000 for immunoprecipitation (IP)),
rabbit anti-Myc (cat#2272, CST, 1:1,000 for WB),
rabbit anti-HA (cat#3724, CST, 1:1,000 for WB, 1:800 for IF, 1:800 for IP),
mouse anti-Ubiquitin (cat#14049, CST, 1:1,000 for WB),
rabbit polyclonal anti-Bip (antiserum, 1:1,000 for WB),
HRP-conjugated goat anti-rabbit IgG (cat#AP132, Millipore, 1:2,000 for WB),
HRP-conjugated goat anti-mouse IgG (cat#AP124, Millipore, 1:2,000 for WB),
Alexa 555 goat anti-rabbit IgG (cat#A21428, ThermoFisher Scientific, 1:2,000 for IF),
Alexa 488 goat anti-mouse IgG (cat#A11001, ThermoFisher Scientific, 1:2,000 for IF).

Validation

All antibodies were commercially obtained and validated by vendors and published studies (see the website of the manufacturer).
The antiserum of rabbit anti-Bip was validated in the previous work (Gao et al., Current Biology 2018, 28(17):2763-2776.e6.).
mouse anti-Myc (cat#2276, CST),
https://www.cellsignal.com/product/productDetail.jsp?productId=2276
rabbit anti-Myc (cat#2272, CST),
https://www.cellsignal.com/product/productDetail.jsp?productId=2272
rabbit anti-HA (cat#3724, CST),
https://www.cellsignal.com/product/productDetail.jsp?productId=3724
mouse anti-Ubiquitin (cat#14049, CST),
https://www.cellsignal.com/product/productDetail.jsp?productId=14049
HRP-conjugated goat anti-rabbit IgG (cat#AP132, Millipore),
https://www.merckmillipore.com/CN/en/product/Goat-Anti-Rabbit-IgG-Antibody,MM_NF-AP132
HRP-conjugated goat anti-mouse IgG (cat#AP124, Millipore),
https://www.merckmillipore.com/CN/en/product/Goat-Anti-Mouse-IgG-Antibody,MM_NF-AP124
Alexa 555 goat anti-rabbit IgG (cat#A21428, ThermoFisher Scientific),
https://www.thermofisher.cn/cn/zh/antibody/product/Goat-anti-Rabbit-IgG-H-L-Cross-Adsorbed-Secondary-Antibody-Polyclonal/A-21428
Alexa 488 goat anti-mouse IgG (cat#A11001, ThermoFisher Scientific),
https://www.thermofisher.cn/cn/zh/antibody/product/Goat-anti-Mouse-IgG-H-L-Cross-Adsorbed-Secondary-Antibody-Polyclonal/A-11001

Animals and other research organisms

Policy information about studies involving animals; ARRIVE guidelines recommended for reporting animal research, and Sex and Gender in Research

Laboratory animals

Balb/c inbred mice and ICR outbred mice (female, 6–8 weeks old) were housed in Xiamen University Laboratory Animal Center and kept at 22–24°C under a 12:12 light-dark illumination cycle at a relative humidity of 45%–65%.
Larval of the Anopheles stephensi mosquitoes (Hor strain) were raised at 28°C and 75% humidity under a 12:12 light-dark condition in a standard insect facility. Adult mosquitoes were fad with 5% sucrose solution and reared at 24°C.

Wild animals

No wild animals were involved in this study.

Reporting on sex

Female mice were used for parasite infection of the rodent malaria parasites; three to five mice per cage were arranged. For generate Plasmodium yoelii genetic cross, female mosquitoes were used.

Field-collected samples

No samples were field-collected.

Ethics oversight

All animal experiments were performed according to protocols approved by the Animal Ethics Committees at Xiamen University (XMULAC20190050).

Note that full information on the approval of the study protocol must also be provided in the manuscript.
