# Supplementary material for: Deaggregation of mutant Plasmodium yoelii de-ubiquitinase UBP1 alters MDR1 localization to confer multidrug resistance
Source: Nat Commun. 2024 Feb 27;15:1774. doi: 10.1038/s41467-024-46006-3 (PMC10899652; doi:10.1038/s41467-024-46006-3)

Uncropped blots (for Fig. 3e, 3f, 3g, 4b, 5e, 5f, and 5g)

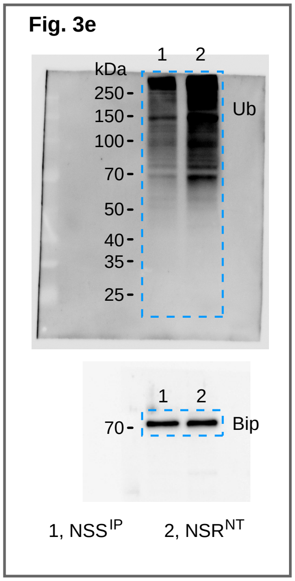

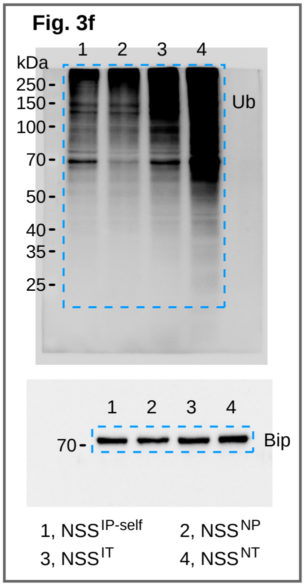

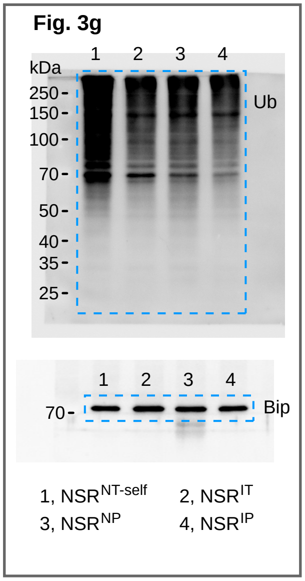

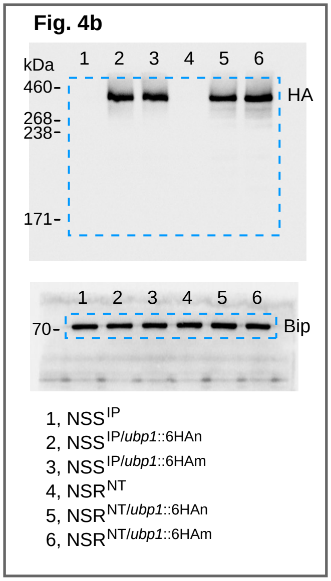

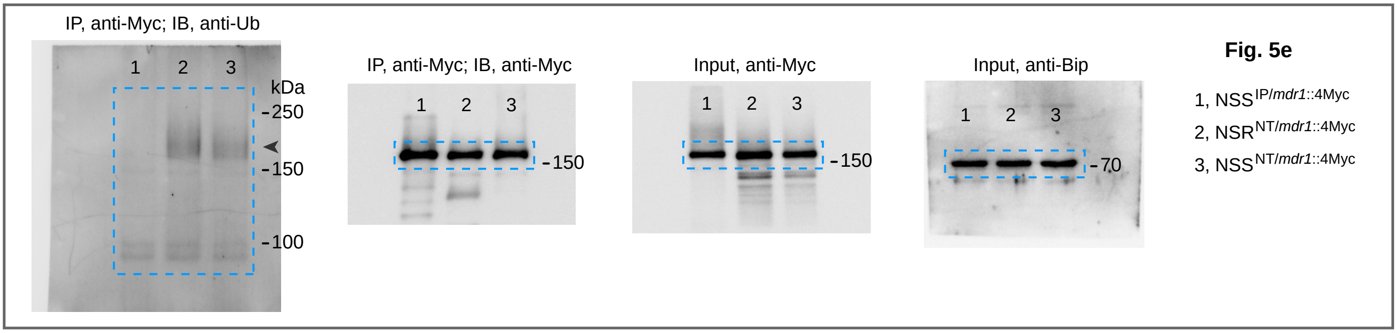

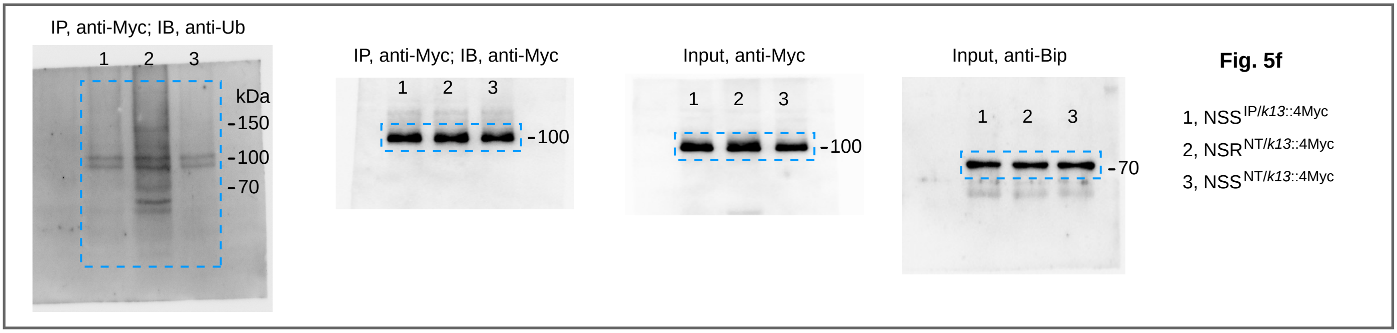

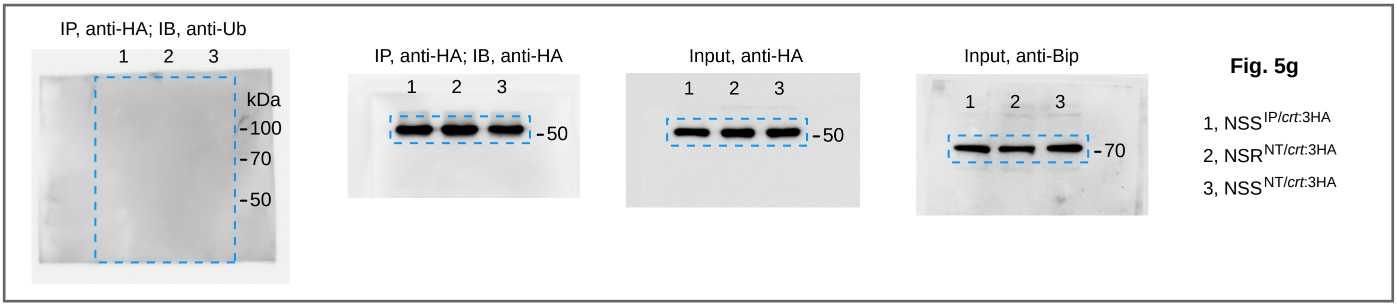

Supplement: Supplementary file 10 — Source Data [file 41467_2024_46006_MOESM10_ESM.zip › Uncropped gels.pdf]
